# What is Family and Group Conferencing for adults? Part 1: Characterising the model and methods of enquiry

**DOI:** 10.3310/nihropenres.13811.2

**Published:** 2025-06-06

**Authors:** Sharanya Mahesh, Jerry Tew, Mary Mitchell, Kar-Man Au, Vicky Nicholls, Abyd Quinn Aziz, Miranda Johnson, TK Vincent

**Affiliations:** 1 University of Birmingham School of Social Policy, Birmingham, England, UK; 2 School of Social and Political Science, University of Edinburgh, Edinburgh, UK; 3 Cardiff University School of Social Sciences, Cardiff, Wales, UK; 4 Independent lived experience researcher, London, UK; 5 Royal Borough of Kensington and Chelsea / Westminster City Council, London, UK; 6 Fulcrum Family Services, Bromley, UK

**Keywords:** Family Group Conferencing, strengths-based practice, person-centred care, adult social care, social work, mental health

## Abstract

**Background:**

Family and Group Conferencing (FGC) is a relatively new strengths-based approach applied to adults needing social care and mental health support in the UK. Although the approach is well established in children’s services in the UK, few services currently offer FGCs to adults; therefore, there is limited evidence regarding FGCs in the adult services context in the UK. This study aims to fill this gap by examining how FGCs work and what differences they can make in people’s lives.

**Methods:**

This paper (Part 1) addresses the first of two related research questions, aiming to characterize the practice model(s) that pertain to the context of adults’ FGCs as currently offered. We employed a mixed methods research design drawing on data from both, previous literature as well as current practice by undertaking a comprehensive literature review, national survey and stakeholder interviews with current services and a deliberative forum involving a range of stakeholders pertaining to adult FGCs.

**Results:**

Although there is some variation in the practice model(s) offered by services, the overall approach is underpinned by a relatively consistent set of values and principles, although these are often implicit. The FGC offers a staged approach that enables people and their networks to take greater control over their support arrangements. It is seen as an appropriate service offer across all adults and mental health services with the potential to result in a range of positive outcomes (as will be discussed in Part 2).

**Conclusion:**

Central to achieving this is flexibility within the service offer to accommodate the social and cultural needs of the individual and their network, the independence of the FGC coordinator, the necessity of sufficient preparation for all participants, and rapport building in advance of the Conference.

## Plain language summary

Family and Group Conferencing (FGC) is a relatively new strengths-based approach applied to adults needing social care and mental health support in the UK. The approach is well established within children’s services however, few services currently offer FGCs to adults and therefore, there is limited evidence regarding how FGCs work and what difference it can make in peoples’ lives in the UK. This paper (Part 1) sets out to fill this notable gap and address the first of the two related research questions- aiming to characterise the practice model(s) that pertain to the context of adults’ FGC currently offered. The study employs a mixed methods research design, drawing on a literature review, national survey, semi-structured interviews and a deliberation forum to inform our findings. There is some variation in the current practice model (s) offered by services, but the overall approach is underpinned by a relatively consistent set of values and principles although, these are implicit. FGC offers a staged approach which can enable people and their networks to take greater control over their support arrangements. It is seen as an appropriate service offer across all adult and mental health services with the potential to result in a range of positive outcomes (as will be discussed in Part 2). Central to achieving this is flexibility within the service offer to accommodate the social and cultural needs of the individual and their network, the independence of the FGC coordinator, the necessity of sufficient preparation of all participants, and rapport building in advance of the Conference.

## Introduction

Over the last decade, policy changes across the four nations in the UK have seen a shift towards person-centred or strengths-based working to fulfil adult social care duties. In England, the Care Act 2014, the leading policy framework for adult social care, has positioned strengths-based practice at the forefront of practice requirements ( Mahesh *et al.*, 2024), which is similar to the Social Services and Wellbeing (Wales) Act of 2014. Amongst other practical developments, Family and Group Conferencing (FGC) is one such emerging strengths-based approach ( Price *et al.*, 2020) within adult social care and mental health services. This approach has parallels with other inclusive and empowering approaches to planning and decision making, such as Open Dialogue in mental health ( Seikkula *et al.*, 2003), Person Centred Planning ( Sanderson & Duffy, 2007), Circles of Support ( Community Circles, 2022) and processes for Shared Decision Making (SDM) in health settings. For instance, the stages of SDM are reflected in FGCs ( Ramon, 2021) however, a key difference remains in relation to the central person and their network being able to reach their own understanding of what may work in the absence of professionals. This places FGC in a unique position to enable individuals to take control of their process and encourage outcomes that makes most sense to them. 

Originating in New Zealand, FGC was first introduced in the 1980s to address the disproportionate number of Māori children and young people needing support and care ( Brown, 2003). Since FGC’s formalisation in New Zealand’s child welfare legislation in 1989, a number of countries have introduced FGC within children and adult social care services. The first ever account of FGC in the UK dates back to the early 1990s, when Family Rights Group, a charitable organisation, attempted to establish the model across children’s services ( Brown, 2003). Since its first pilot initiative in England and Wales in 1993, a recent UK wide survey suggests that nearly 79% (167) of local authorities in the UK offer FGC as part of their children’s social service offer ( Wood *et al.*, 2022).

While a systematic review has found inconclusive evidence regarding FGC outcomes for children ( Nurmatov *et al.*, 2020), a more recent Randomised Controlled Trail has demonstrated positive results in relation to a number of outcome measures, with significantly fewer children going into local authority care and, for those that did go into care, this being for a shorter duration ( Taylor *et al.*, 2023). Cost efficiencies were also reported, with local authorities receiving a net benefit of £960 per child in the first year of entering pre-proceedings for an FGC. 

 However, FGC remains a relatively new approach within UK adult social care and mental health services despite some international evidence suggesting positive outcomes. There also remains a lack of systematic evaluations of the implementation and effectiveness of FGC in the UK context ( Górska *et al.*, 2016; Manthorpe & Rapaport, 2020; Parkinson *et al.*, 2018). By employing a realist synthesis approach, this study aims to fill a notable gap in research evidence to better understand how FGC is delivered in practice and what outcomes may be achieved.

FGC is often described as an innovative and inclusive approach that empowers individuals to take control of their lives ( Górska *et al.*, 2016). Unlike other social care meetings, it is a voluntary, citizen-led, participatory decision-making approach based on the assumption that family and social networks can be a powerful tool in supporting people to make and implement plans relating to their care and support ( Ramon, 2021). It is based on the premise that individuals and members of their relational network are experts of their situation ( Parkinson *et al.*, 2018) and are, therefore, best placed to make decisions.

Currently, there is no agreed definition for FGC with adults, but within the context of children and families, it has been described as:

‘Decision making meetings in which plans are constructed by a family (including extended family members and friends) to address identified child welfare concerns and ensure a child’s future safety and well-being’ ( Owens *et al.*, 2021).

FGCs are often deployed at the ‘pre-proceedings’ stage to see whether it may be possible to avoid children being taken into care by the local authority, where there may be serious concerns regarding their welfare.

While there are strong parallels with the practice model that has emerged for children and families, this definition cannot be straightforwardly transferred to the emerging use of FGC as a practice approach for adults who may face challenges or difficulties. In the UK, the legal context for adult social care is very different from that for children; therefore, there is little equivalence to the use of FGC to avoid the need for legal proceedings that may be taken to remove children from parental responsibility. Safeguarding may or may not be an issue, but the legal and policy contexts for adults’ FGCs tend to be much more voluntary, with people coming together to make a plan whereby a person may be both better protected and better supported to live the sort of life that they want to lead.

Another key difference is that the network of ‘people that matter’ to an adult may or may include family members and may equally include friends, neighbours, and anyone else that may be identified as relevant to the individual ( Parkinson *et al.*, 2018). For this reason, we have proposed, in consultation with practice colleagues, the insertion of the word ‘and’ between Family and Group, so as to offer a more inclusive naming of the practice as Family *and* Group Conferencing for adults.

From our interviews with stakeholders, a widespread concern emerged that FGCs were often not well understood by wider services – for example, being misperceived as some form of mediation or family therapy service, sometimes attributing this confusion to the term ‘family’ in the title. Therefore, there is a strong need from the practice field of adults and mental health services for greater clarification of what FGCs are and are not. However, as will be discussed in more detail later, there are strong indications that FGCs may achieve more than just the practical task of making key decisions regarding safety, care, or support. For example, Johansen (2014) argues that in the context of mental health, FGC should be regarded as a ‘recovery model’ rather than just a decision-making model. Studies have often highlighted other sorts of positive outcomes from FGC, with significant changes in how the closer and wider relational systems around the person may come to operate. Common outcomes generally include empowerment of individuals and their network members ( Parkinson *et al.*, 2018), improved resilience of network members ( Ramon, 2021) and a greater sense of community and self-worth ( Malmberg-Heimonen, 2011). Although there is limited evidence, small-scale studies in the UK have reported FGC’s potential of FGC to result in cost efficiencies ( Marsh & Crow, 1998).

This Paper reports findings from the first part of a wider research study *Family Group Conferencing in adult social care and mental health: exploring how it works and what difference it can make in people’s lives* which has been funded by the National Institute for Health and Care Research (NIHR). This Paper (Part 1) addresses the first of two related research questions, aiming to characterise the practice model (or models) that pertain to the context of adults’ FGCs as currently offered. The linked Part 2 Paper will examine the current understanding of how the process may work, what sort of outcomes may be achieved, and what contextual factors may influence whether such outcomes are achieved, which may be termed an initial Programme Theory for adults.

For clarity and simplicity, we used the following terminology in the subsequent discussion:

- **FGC** denotes either Family and Group Conferencing or Family and Group Conference
- **Coordinator** denotes an independent person, whose role is to facilitate people coming together and making a plan.
- **Central person** denotes the adult with support needs with whom, and around whom, the FGC process is organized.
- **Network** denotes family members, friends, and others invited to participate in the FGC.
- **Private time** denotes the part of the Conference when the central person and network members are in charge of formulating their plan. The coordinator and invited practitioners usually leave a Conference for this purpose.

## Methods

### Patient and public involvement

An important aspect of this study is the involvement of individuals with lived experience in a number of different capacities. To ensure that we have captured all perspectives on our data, the study employed two peer researchers with lived experience in the research team to support our literature review, interviews, and subsequent analysis. In addition, the study was guided by a panel of four individuals with lived experience of an FGC, either in the capacity of being the central person or a network member to provide advice, guidance, and challenge. In particular, our lived experience advisory panel (LEAP) was instrumental in shaping our survey and interview questions and in providing feedback on our findings. Through the deliberative forum, we were able to gather extended feedback from other individuals with a lived experience of an FGC, which enabled us to capture a wider range of perspectives and validate our findings. Further details on the contribution of individuals with lived experience is provided in the following sections.

***Research methods***

The aim of this study was to examine how FGC for adults and mental health is being delivered in the UK and, in particular, how practice model(s) may be characterised (the focus of this paper) using a realist approach ( Pawson & Tilley, 2004), which may be the programme theory that underpins this (the focus of the Part 2 Paper to follow). We sought to achieve a comprehensive understanding through an integrative approach, drawing together findings from an analysis of the international literature, a national online survey, interviews with selected stakeholders, and a deliberative forum comprising a cross-section of informants from the field.

**Figure 1.**
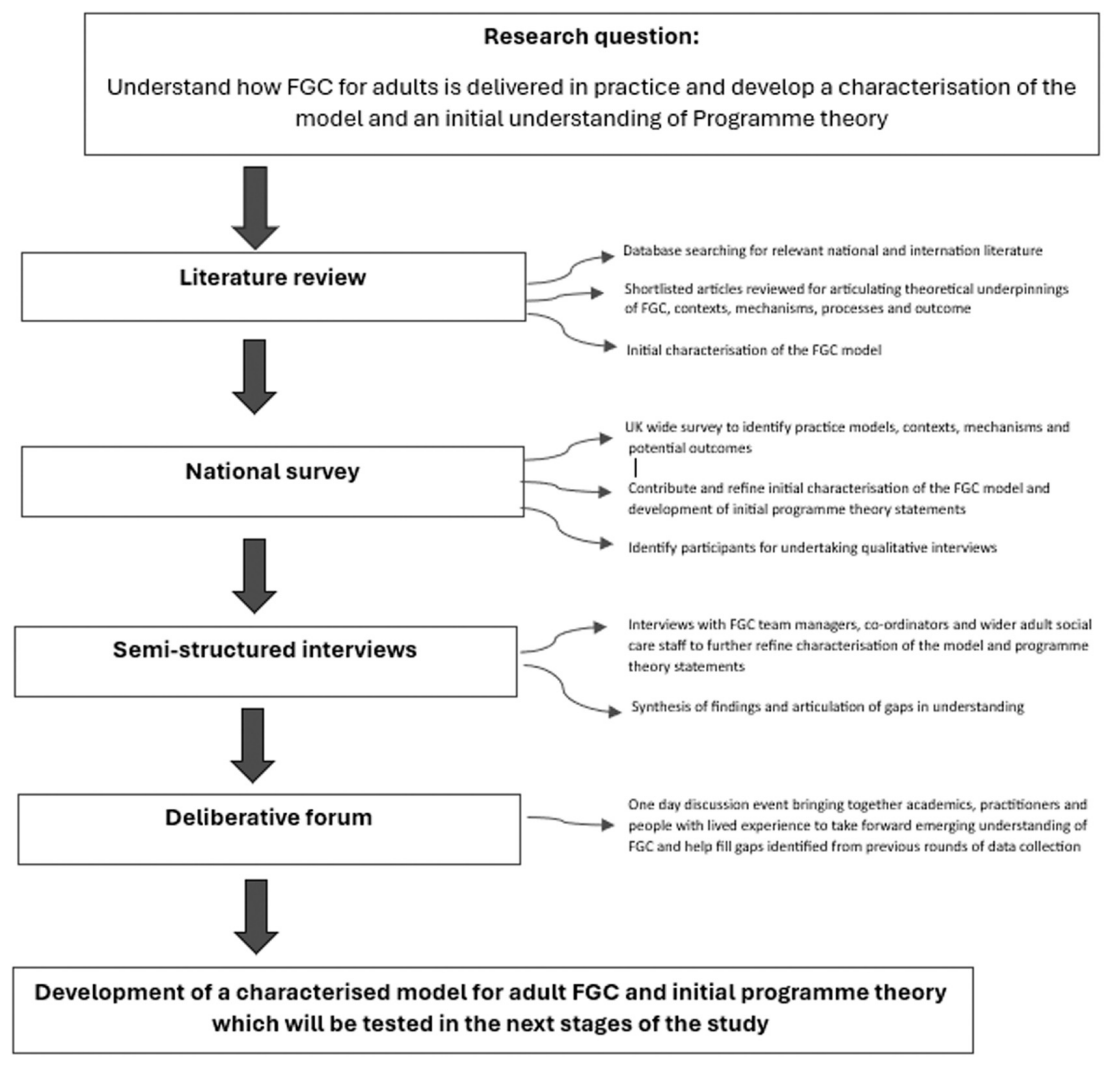
Schematic diagram of methods employed.

Out of this process, certain key questions emerged where there remained a lack of clarity or gaps in understanding, and these questions were then put forward for discussion at a Deliberative Forum. Putting this together provided a basis for an initial formulation of (a) how adults’ FGC practice model(s) may be characterised and (b) what key context-mechanism-outcome configurations (CMOCs) may constitute their underpinning programme theory. This initial formulation was used to inform subsequent evaluative research conducted at the selected case study sites. Figure 1 provides a schematic diagram of the methods employed.

**Literature review**

Drawing on international literature, this review aims to provide a narrative synthesis of key themes relating to adult FGCs with a particular focus on how the practice model may be described and defined, its underpinning values and principles, and for whom it was considered a suitable approach. In the early stage of the review process, it was considered whether to include literature relating to FGC in children’s services, where practice may be significantly different but might nevertheless provide useful insights into how FGC for adults might be working. However, given the volume of the literature available relating to children’s FGCs, a decision was made to focus the review on literature relating to FGC practice generically or that which specifically relates to adults and mental health services, while also drawing on some theoretical perspectives that have been proposed in relation to FGC practice with children and families.

To identify relevant literature, database searches were conducted in APA PsycINFO- OVID, Applied Social Science Index and Abstracts (ASSIS), Social Services Abstracts, and ProQuest Social Science Journals (Social Science Database). Each database was searched for word combinations of family group conferencing/family group decision-making AND

*Adult, *social work, *mental health, *outcomes, *FGC theory, *empowerment, *evaluation, *community mental health, *public mental health, *quality of life, *resilience, *social support, *restorative justice, *recognition, *social capital

**Figure 2.**
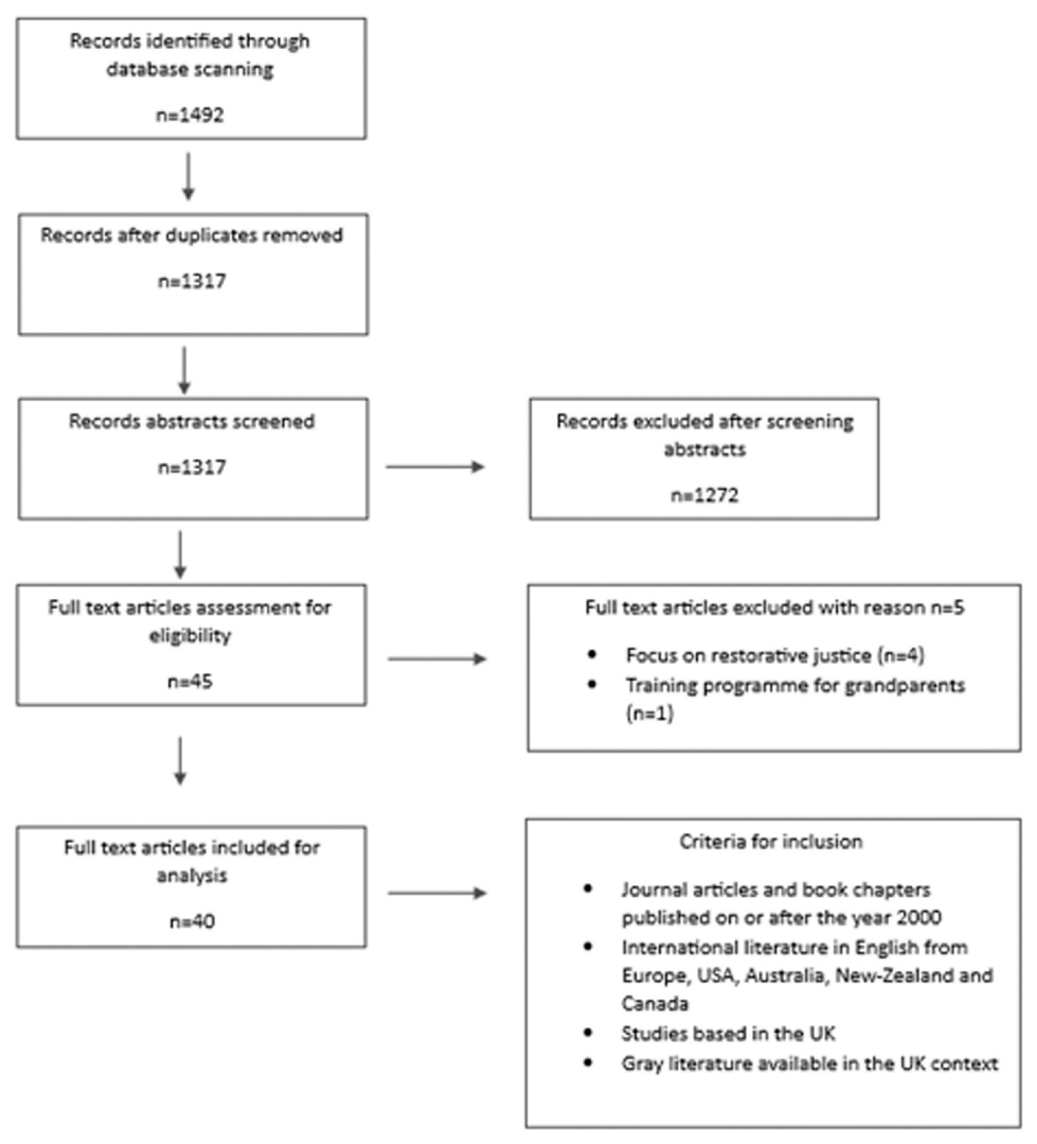
Breakdown of study selection.

In addition, multiple combinations of the above search terms were used to ensure the inclusion of all or nearly all relevant articles pertaining to our topic. For example, we combined terms such as ‘family group conferencing’ AND ‘adult’ AND ‘mental health’ NOT ‘child. ’ We also conducted a search for grey literature across UK based professional and policy agencies. Initially, upon identifying relevant documents, we used snowballing to further identify organisations/resources related to adult FGCs. Our inclusion criteria were as follows: (a) all articles, book chapters, and gray literature produced between 2000 and 2023; (b) national and international literature emerging from the UK, Europe, USA, Australia, New Zealand, and Canada; and (c) literature that focuses on any or all contexts, mechanisms, and outcomes relating to adult FGCs. The selection of appropriate literature was based on the Preferred Reporting Items for Systematic Reviews and Meta-Analyses (PRISMA) guidelines (see Figure 2 below).

The final selection of literature is presented in Table 1 (below). Zotero referencing software ( https://www.zotero.org/) was used to manage this information in a database that was available to all members of the research team.

**Table 1.** Summaries of the included literature.

Authors	Title	Date of Publication	Summary of abstract
de Jong and Schout	Breaking through Marginalisation in Public Mental Health Care with Family Group Conferencing: Shame as Risk and Protective Factor	2013	Adopting a case study approach, this study examined whether FGCs are valuable for service users in a public mental health care setting in Netherlands as a means to generate social support, prevent coercion and elevate the work of professionals. Findings suggest that shame and fear of rejection were the primary reasons for service users to avoid contact with their social network, resulting in isolated and marginalised living situations. Shame was also reported as a powerful engine for marginalised circumstances. An FGC was seen as a forum to discuss feelings of shame and generate support from social networks. The study also concludes that limited or broken networks are not a contraindication but a reason for organising FGCs.
Schout and de Jong	Collecting feedback as a tool to reduce care paralysis: something for family group conferencing coordinators?	2016	Drawing on empirical and theoretical findings, this paper considers the possibility of collecting feedback as an effective way of contributing positively to the relationship between FGC coordinators and service-users. This study is set in the Dutch primary mental health care context where service-users have little faith in professionals and demonstrate hostility in engagement. Findings indicate the importance of feedback theory for FGC coordinators in enhancing trust and engagement.
de Jong, Schout, Meijer, Mulder and Abma	Enabling social support and resilience: outcomes of Family Group Conferencing in public mental health care	2015	This study examined outcomes from 41 conferences held within a primary mental health setting in norther Netherlands. Service users referred for a conference mostly had a limited network and few recourses from whom little support could be expected. Deploying t-tests and multilevel analyses on responses from 245 participants, findings suggest significant positive changes (from prior to after the conference) on three measures- (a) social support (b) resilience and (c) living conditions.
de Jong and Schout	Evaluating Family Group Conferencing: Towards a meaningful research methodology	2018	This study seeks to examine the theory on programme evaluation as an effective research methodology to study FGC. Although a RCT is the golden standard, it only provides a partial understanding of the complexities experienced by families. The study highlights that the context conferences are challenging as it is where the lifeworld of families constantly interacts with the system world of professionals and is characterised by multiplicity, polyvalence and interference. The authors conclude that the methodology used to examine the efficacy of FGC should meet this ‘interplexitiy’.
de Jong, Schout, and Abma	Examining the Effects of Family Group Conferencing with Randomised Controlled Trials: The Golden Standard?	2015	In this critical commentary, the authors argue against recent criticism of FGC evidence especially when alternative methods to RCTs have been employed to research FGC. The study makes a comparison with how RCTs are conducted studies. Findings suggest that a RCT design cannot control conditions in the social reality of families the impact of unintended side effects. The study concludes that questioning the qualitative and evaluation methods that have been used so far to examine the outcomes of FGC is justified, and neither is there any reason to be uncritical towards the evidence that RCTs might provide.
Ramon	Family Group Conferences as a Shared Decision-Making Strategy in Adults Mental Health Work	2021	This article provides a narrative review of existing empirical research about FGC in the context of adult mental health. In addition, two community case studies consisting of videos of a mother experiencing mental ill health and a daughter are analysed in terms of their subjective experience of the FGCs they were involved in and examines the process and outcomes of FGCs. Findings suggest a promising strategy for SDM and demonstrates a high level of satisfaction from participating in the FGC meeting, while the evidence pertaining to the outcomes is inconclusive. The observed gap between the satisfaction from the process of FGC by the participants vs. the inconclusive outcomes relates to the implementation phase, in which the decisions made by the family are tested. There is a need for further exploration of its implementation process, evaluative methodology and methods.
de Jong and Schout	Family group conferences in public mental health care: An exploration of opportunities	2011	This study reports on an exploratory study on the applicability of FGC in public mental health care. Findings suggest that there are six reasons to start FGC pilots in public mental health care (a) professionals in public mental health care setting need to deal with service-users who are not motivated in seeking help and FGC can yield support even in the absence of the service-user (b) FGC may complement the repertoire of treatment options between voluntary help and coercive treatment (c) clients in public mental health care often have a limited network and FGC can expand and/or restore relationships (d) conferences could succeed both in a crisis and in other non-critical situations (e) service users with negative experiences with care agencies might be inclined to accept a conference because professionals have limited input (f) the social network could elevate the work of professionals.
de Jong, Meijer and Schout	Family Group Conferencing as a Catalyst for Recovery and Ownership in Mental Health	2018	This study employed a qualitative case study framework to examine 41 FGCs held within a public mental health care setting in Netherlands. This article highlights two case portraits and gives insight into how ownership was restored and sheds light on the service users’ recovery process. The authors conclude that FGC is seen as a promising tool to shift the attention from disorders and inabilities to capacities and the rediscovery of social resources.
Blundell, Clare & Clare	Family Group Conferencing as an Additional Service Response to the Abuse of Older People in Australia	2021	This paper presents the core processes of FGC, including the quality of the established evidence base for its use with older people. The limitations and caveats associated with this approach are explored, and a way forward is proposed to explore the utility and suitability of FGC for adults in Australia in response to some types and severities of abuse and mistreatment. Findings indicate that abuse of older people is complex and under-reported, and many older people choose to take no action as a large proportion of perpetrators are family members. FGC may enhance secondary-level service responses to abuse, mobilising the older person’s protective networks, and reducing the risk of abuse.
Metze, Abma and Kwekkeboom	Family Group Conferencing for older adults: Social workers’ views	2019	This study examined reasons for social workers’ reluctance to refer older adults to FGC in Netherlands. Employing an exploratory methodology, this mixed methods study concluded that social workers were positive about FGC but were hesitant for reasons – they were already working with their service-users’ social networks, fear of losing control over the care process, challenges associated with motivating service users. The study also noted reluctance from service-users due to fear of losing self-mastery and not wanting to burden their social networks. The authors conclude that implementing FGC in elderly care can be complicated and slow process partly because social workers have little experience with FGC. It may be useful to experiment with alternatives to FGC, for example, by focusing less on family networks and more on reciprocity.
Meijer, Schout and Abma	Family Group Conferencing in Coercive Psychiatry: On Forming Partnership Between the Client, Social Networks and Professionals	2019	This study examines the process and outcomes of FGC organised for service-users who were at the risk of coercive treatment in psychiatry in Netherlands. Findings suggest that service-users felt a sense of ownership and control over their situation and that agreements made with loved ones were more serious than those made with professionals. FGC can widen the circle of support by involving family and friends who are generally willing to support. Disclosing shameful feelings and embracing vulnerability, changing attitude (of letting go on control) and facilitation abilities of professionals are crucial in forming a partnership.
Górska, Forsyth, Prior, Irvine and Haughey	Family group conferencing in dementia care: an exploration of opportunities and challenges	2016	This qualitative study aimed to evaluate the impact of the pilot FGC service, delivered to people with dementia and their families, in terms of the experience of care provision by families and care professionals involved in the project. FGC was perceived as having positive impacts on service users, their families, service providers and the wider culture of care. However, participants identiﬁed a number of challenges related to service implementation-challenges of coherently articulating the needs of a person with dementia, difficult family dynamics and balancing involvement of professionals.
De Jong, Schout, Pennell and Abma	Family Group Conferencing in public mental health and social capital theory	2015	This qualitative study reports findings from 18 FGCs held in a public mental health setting in Netherlands which did not succeed as issues emerged during preparation or because a plan was never reached or fully implemented. Interviews indicate that conferences were often held as a last resort, in situations where professional care had already failed. The intended goal was not achieved because support from the social network was insufficiently mobilised and service users felt helpless about their situation. The study concludes that a single conference was insufficient to break through a sense of inadequacy and paralysis. Social capital theory points to the necessity of not only renewing informal networks (‘strong ties’) but of expanding networks through connecting public mental health care service users to paid and volunteer work (‘weak ties’).
Schout, Meijer and de Jong	Family Group Conferencing—Its Added Value in Mental Health Care	2017	This study aimed at determining the applicability of mobilising help from social networks of people with psychiatric problems. Specifically, this discursive paper sought to address ‘what FGC adds to the existing methods that aim to reduce coercion in mental health care and promote inclusion’. Findings suggest that there is a wider applicability of FGC, even outside the framework of coercive care. This was based on a person’s right to make a plan on their own, ability to address complex sets of problems experienced by this group, people’s desires to change for their relatives and not professionals and FGC creates an opportunity to realise relationships. Where there were difficulties, professionals must act to treat psychiatric conditions before enlisting FGC and family driven strategies should first reserve space for professional driven interventions.
Metze, Abma and Rick Kwekkeboom	Family Group Conferencing: A Theoretical Underpinning	2013	This study aims to provide a theoretical basis for FGC by examining how the concept of empowerment can be linked with the basic assumptions underlying the FGC. Can making a plan of their own indeed help to empower people and if so, how does the process of empowerment proceed? Empowerment is often mentioned as a goal of the FGC, but authors are not unanimous when it comes to the operationalisation of empowerment, especially on the relational level of the person in his or her social context. In the article, the authors use the concepts of relational autonomy and resilience to conceptualize empowerment on the relational and individual level.
Tew, Nicholls, Plumridge and Clarke	Family-Inclusive Approaches to Reablement in Mental Health: Models, Mechanisms and Outcomes	2017	Framed within a realist evaluation and adopting a comparative case study approach, this paper examines ‘whole family’ models of practice and how these may (or may not) contribute to the reablement of people with mental health difficulties. Specifically, the study explored the relationships between contexts, mechanisms and reablement outcomes. An analysis of interviews with twenty-two families highlighted with different starting points and routes, engaging with whole families may lead to the construction of a secure and empowering base from which service users may reconnect with wider social worlds.
Fisher, Mooney, and Papworth	FGCs and adult social care	2018	This book chapter addresses the use of FGC in adult social care. Professional guidance and introduction of policy concepts such as personalisation, wellbeing and person-centred approaches have been supportive of FGC. Indeed, the foundations are in place for FGCs to become an embedded part of adult social care policy and service delivery for vulnerable adults. The chapter then highlights the evidence base for FGC particularly highlighting the potential of FGC to engage families for the benefit of vulnerable adults. The chapter concludes that for FGC practice to develop in the UK, it is important to roll out the FGC service into a wider range of adult settings such as adult mental health, prisoners and homeless people.
Jenkins	For the Common Good: Rob van Pagée on Family Group Conferencing in the Netherlands	2010	This article provides an overview of the FGC model as implemented in the Netherlands. The paper highlights that FGCs can be applied in many diverse settings such as child welfare, with people experiencing domestic violence, impeding eviction and within wider settings where there may be conflicts in neighbourhoods or communities. The model is based on the foundation of sharing power with the public and employing citizens to carry out conferences rather than professionals. FGC has been able to provide family members a better understanding of the person’s situation and realise what matters to them.
Hardy	How social workers can use family group conferences in adults’ services	2017	This blog piece provides an overview of FGC with adults. FGC is reported to be applicable to a wide range of issues – deliberate abuse, neglect, self-neglect or prevention, dementia support, transition from children to adult’s services and best interest decision-making. The blog highlights that empowerment and people being experts of their situation are the key principles underpinning FGC. FGC in adults has three distinct stages – information sharing, private family time and agreeing the plan.
Metze	Independence or interdependence? A Responsive Evaluation on Family Group Conferencing for Older Adults	2015	Employing a case study approach, this study aimed at examining how FGC can help older adults retain and/or enhance their relational empowerment and what factors at the level of social workers and older adults influence the implementation of FGC. Findings suggested that FGC for older adults can be successful in enhancing relational empowerment if – professionals actually offer it to service users, older adults are open to sharing their problems with their network, older adults have sufficient level of resilience and relational autonomy, a diverse and capable social network, problems are related to internal factors of the older adults, and not caused by external factors such as generational poverty or heavy informal care duties and expectations of older adults correspond with their network and welfare state.
Hobbs and Alonzi	Mediation and family group conferences in adult safeguarding	2013	This study reports on a literature review on the use of mediation and FGC in the context of adult safeguarding in the UK. It explores how such ‘family-led’ approaches to adult safeguarding fit with the wider agenda of personalisation and empowerment, including the Mental Capacity Act 2005. Findings suggest that mediation and FGC are inclusive processes that enable people to explore choices and options in a supportive environment, assuring maximum possible independence and autonomous control over basic life decisions. When used appropriately, both approaches can be a valuable response to safeguarding concerns, promoting choice and control at the same time as protecting people from risk of abuse and harm. However, there are few robust evaluation studies currently available and no systematic research studies were found on cost-effectiveness.
de Jong, Schout and Abma	Prevention of involuntary admission through Family Group Conferencing: a qualitative case study in community mental health nursing	2014	Employing a naturalistic qualitative case study approach on one case identified from a larger set of data of 41 conferences held in a primary health care setting in Netherlands, this study aims to understand whether and how FGC might contribute to the social embedding of service users with mental illness. Findings highlight that to prevent involuntary admission to a psychiatric ward of a man with schizophrenia, neighbourhood residents requested for FGC between themselves, the person’s family and the mental health organisation. Nine months after the conference, liveability problems in the neighbourhood had been reduced and coercive measures adverted. The conference strengthened the community and resulted in a plan countering liveability problem.
Johansen	Psycho-Social Processes and Outcomes of Family Group Conferences for Long-Term Social Assistance Recipients	2014	The aim of this study was to explore which psycho-social processes and outcomes does FGC generate for long-term social assistance recipients. Fifteen Norwegian social assistance recipients who had arranged FGCs were interviewed and five were observed. Findings highlight that a key motivation to arrange an FGC was relation-based and correspondingly, the most important outcome was improved relationships through respectful communication. The findings indicate that the FGC may have the potential to strengthen the sense of community and self-worth among social assistance recipients.
Manthorpe and Rapaport	Researching Family Group Conferences in Adult Services: Methods Review	2020	As a methods review, this review outlines the methods used to obtain the evidence about FGCs, commenting on the advantages of diﬀerent methods and their disadvantages. The report also considers the theoretical underpinnings of FGC and international research evidence regarding use and effectiveness of FGC. The report highlights the use of main research methods to study adult FGCs but a significant gap regarding cost-effectiveness remains. Further, few studies have collected data about the medium- to long-term outcomes for the people concerned to consider if promising outcomes are sustained and individuals’ wellbeing enhanced.
de Jong and Schout	Researching the Applicability of Family Group Conferencing in Public Mental Health Care	2013	This research note aims to examine the application of FGC in mental health care in a public mental health care setting in Netherlands. The study aspired to be undertaken with the assumption that FGC promotes involvement, expands and restores relationships and generate support. The aim of the study is to provide an answer to the question of whether FGC is an effective tool to generate social support, to prevent coercion and to promote social integration in public mental health. As part of the preparation stage, professionals were trained in strengths-based working, mainly to focus on the strengths and resources of service users.
Markel-Holguin	Sharing Power with the People: Family Group Conferencing as a Democratic Experiment	2004	This article reviews children FGCs and adaptations of the model to different contexts, from the perspective of democratic practice and through the lens of responsive regulation. The study suggests that considering FGC in a democratic and responsive regulation context provides a theoretical construct to mainstream this practice and challenges years of professional domination. It demands new strategies for engaging the citizen as active participants in creating a community-based and responsively regulated system that protects children and supports families. The mainstreaming of family group conferencing lies in a collective understanding of this model as a practice that supports the pillars of democracy, one that promotes self-regulation, and one that fosters responsive regulation by encouraging differential response to families through individualising plans to more closely meet identified needs.
Metze, Kwekkeboom and Abma	The potential of Family Group Conferencing for the resilience and relational autonomy of older adults	2015	Employing a case study approach, this article reviewed two FGC cases of older adults to study the appropriateness of the concepts of resilience and relational autonomy in their FGC. Findings indicate that compassionately interfering social contacts, showing respect for the older person's needs and wishes gave older adults an impulse to take action to solve their problems. The person’s motivation and willingness to ask for her seemed essential to foster behavioural change. Contextual factors such as the nature of the problem, involvement and capacity of the social network were also determining factors.
Schout, van Dijk, Meijer, Landeweer and de Jong	The use of family group conferences in mental health: Barriers for implementation	2017	This qualitative study examined situations and circumstances in which FGC may (not) be useful. Barriers included (a) the acute danger in coercion situations, the limited time available and control and risk aversion in mental health care (b) the severity of the mental state of clients leading to difficulties in decision-making and communication (c) considering FGC and involving family networks as an added value in a crisis situation is not part of the thinking of professionals (d) when clients and their networks are not open to FGC. The study concludes that awareness of FGC barriers helps to keep an open mind in its capacity to strengthen relationships between clients, networks and professionals.
Bredewold and Tonkens	Understanding Successes and Failures of Family Group Conferencing: An in-depth Multiple Case Study	2021	This study critically reflects under which conditions FGCs may or may not be successful. Drawing on data from four longitudinal cases, interviews with social workers, observations of trainings and a literature review, four conditions for the successful application of FGC was identified- (a) presence of a positive network (b) need for formal care in addition to informal care (c) active preparation against paternalism and humiliation (d) taking service-users reluctance to ask social networks for help seriously.
Malmberg-Heimonen a Johansen	Understanding the longer-term effects of family group conferences	2014	Using a randomised controlled design on 149 Norwegian longer-term social assistance recipients, this study analyses long term effects of adult FGCs in terms of social support, mental health and reemployment. In addition to randomly allocating all participants, 15 interviews were conducted to gain in-depth knowledge around FGC impacts. Despite high rate of participant satisfaction and significant shorter-term effects, the one-year follow-up identified neutral effects from FGC. Qualitative interviews demonstrated that lack of reciprocity in social relationships and lack of follow-up were the main reasons for the stagnation of an initially positive FGC process.
de Jong, Schout and Abma	Understanding the Process of Family Group Conferencing in Public Mental Health Care: A Multiple Case Study	2018	This qualitative multiple case study research aimed to examine the process of FGC in a public mental health care setting in Netherlands. Process dynamics identified were (a) overcoming resistance and breaking through isolation and shameful feelings (b) service users change more likely for network members than professionals (c) role of coordinators are complex (d) professionals who cannot resist the temptation to take over. The study further highlighted that four factors influenced the quality of life of the service user (a) willingness of service user to invite and widen their social network (b) willingness of service user and network to share shameful feelings (c) mutual trust between service users and coordinators (d) professionals reinforce self-direction of the group and prevent service users from falling back into individual care trajectory.
Bishop	What is a family group conference for adults? Brief Guide	2017	This guide provides an overview of about FGC. It suggests that network members can include family, friends, neighbours, local community and anyone else who in involved in providing informal care and support. It emphasises on the independence of the coordinator who is expected to not be involved in any decision making of the person. It highlights three main stages to the process- information gathering, private family time and explaining/agreeing on a plan. The blog concludes by shedding light on the review process following the actual conference.
Metze	"With a little help from my friends": Family Group Conferencing and home-evictions	2007	This study aims at addressing what people would do should they be threatened with home eviction. Experiences of those who were offered FGC were compared to two other target groups to identify conditions supportive of successful implementation of FGC with this service user group. The study highlighted that FGC fits well into the idea of people helping each other however, findings did not always suggest a positive outcome. Although FGC improved support from social networks and increased the confidence and security of service-users, plans were not always followed through sometimes due to the lack of motivation.
Metze, Kwekkeboom and Abma	You don't show everyone your weakness’: Older adults' views on using Family Group Conferencing to regain control and autonomy	2015	This qualitative study aimed to examine existing views and attitudes of older adults concerning the use of FGC, and report on how older adults see the possibility to regain control over their lives using FGC. Resistance towards FGC was mainly due to service-users expecting people to be there for them without an FGC, not feeling ready for FGC, feeling embarrassed of asking for people and having the fear of losing control. The study concludes that for older adults, FGC means losing control and autonomy rather than gaining it. In order for FGC to work for older adults, a relational empowerment model should most likely be focused on reciprocity, peer-to-peer support, and solutions instead of problems.
Valenti	Family Group Conferencing with BME Families in Scotland	2017	This qualitative study explores the use FGC in social work with children and families from black and minority ethnic (BME) backgrounds living in Scotland. Findings suggest that despite evidence that FGC can positively impact people from different cultural groups, BME families do not always have access to FGC due to their lack of awareness and other structural issues such as information about FGC only available in English. For those who have had a conference, it widened their circle of support and increased their participation and commitment to the plan.
Waites, Macgowan, Pennell, Carlton-LaNey and Weil	Increasing the cultural responsiveness of Family Group conferencing	2004	This article describes the model and a culturally competent method for assessing and adapting the model for the African American, Cherokee, and Latino/Hispanic communities in North Carolina. The study emphasises on the importance of listening to and learning from community members and tapping into their strengths to reshape FGC to meet the needs of children and families. In particular, the study reported that families appreciate the opportunity to resolve their own problems, FGC can work if cultural adaptations are used and traditions are respected, coordinators are bicultural and bilingual, FGCs must be held in culturally appropriate settings and ongoing communication and joint problem solving are crucial.
Nygård and Saus	Is Family Group Conferencing a culturally adequate method outside its origin in New Zealand? A Meta synthesis	2019	This meta synthesis of 26 articles examines whether FGC is culturally adequate in indigenous communities. Through systematic and strategic searches, the study explored the existing trends of FGC research in indigenous contexts. Analysis indicates that there is a tendency towards taking the cultural adequacy of FGC for granted. A few researchers question these assumptions, and debate tokenism and colonialism in social work. The authors conclude that implementing FGC in new communities requires foundation in local, cultural context.
Barn and Das	Family Group Conferences and Cultural Competence in Social Work.	2016	Empirical study carried out in London to ascertain the views and experiences of FGC coordinators and managers, located in statutory and non-government organisations, who employed the FGC approach with culturally diverse families. Emphasis on the desirability of ethnic, cultural and religious matching of coordinators to families.
Tapper	Using family group conferences in safeguarding adults	2010	This article explores the effectiveness and value of FGC for older people and considers the usefulness of the approach for all vulnerable adults where there may be safeguarding concerns within the context of personalization and self-directed support

In the process of analysing and synthesising the evidence from the literature, we saw it as important in maximising the validity of our findings that we incorporated the ‘standpoint’ expertise of people with lived experience ( Tew *et al.*, 2006), practitioners, and people from different cultural and ethnic backgrounds, so that the content could be viewed from different perspectives and potentially significant evidence was not missed. To this end, a research team was formed that included two peer researchers with lived experience, two researchers with current practitioner experience, and four with academic backgrounds. Half of the research team was from a minority ethnic background. All researchers held similar responsibilities, including reviewing and summarising shortlisted articles, writing up key points emerging from their designated articles, and taking part in group discussions in which we started to draw together understandings of FGC models and processes as applied in adults and mental health contexts.

Prior to undertaking the analysis, the research team held a series of discussions to determine the areas of interest and the scope of the review. To maintain focus and consistency, a reviewing framework was developed that recorded the (a) reference; (b) theory relating to FGC; (c) contextual factors including who could be offered a service, cultural/community acceptability, and wider service context; (d) processes and mechanisms; (e) outcomes for individual, network, and/or service; and (f) other relevant material, including values, perceptions, and assumptions relating to FGC. All articles were independently reviewed by an academic researcher and a team member with either lived experience or practice background. Upon completion of the dual review process, we realized that an additional round of review was required to comprehensively capture the subtleties of FGC. For this round, each member of the research team was instructed to write additional notes on the overarching themes that emerged from their selection of the assigned literature. This material was collated and further analysed by Mitchell, Mahesh, and Tew to provide a comprehensive thematic analysis of the literature and subsequently developed into an initial characterisation of the FGC model for adults and program theory statements.

**National survey**

A national online survey was conducted alongside a literature review. The aim of the survey was twofold: (a) identify adult FGC services in the UK that are in operation or in a set-up phase, and (b) capture an in-depth understanding of the practice model(s) adopted in these services. Survey questions were devised via an iterative process to maximise the opportunity for input and scrutiny from peer researchers and from those with a practice background. A preliminary list of survey questions based on our research questions and initial themes identified from the literature was developed by Mahesh and Tew and circulated to the wider research team for input. Upon receiving feedback, necessary changes were made to reflect their comments and concerns. Feedback included refining the language, changing the ordering of questions, and identifying gaps (for example, including explicit questions regarding the review process and cultural appropriateness of FGC). Version two of the questionnaire was then circulated to the research team for additional review. This generated further minor changes to the language and length of the questionnaire. Questions within our survey were directed towards gaining a coherent understanding of the three phases of the FGC process (preparation, conference, and review), context, and potential outcomes. Online Surveys software, version 2 ( https://www.onlinesurveys.ac.uk/), provided a platform for the survey and was used to assist in the analysis and presentation of the findings.

The survey was conducted between January and June 2023. To create sufficient awareness, we employed a variety of platforms, including dissemination through social media (our project website, X and LinkedIn handles), through our practice partners to share with relevant stakeholders, the Chief Social Worker (adults) and the Principal Social Workers’ Network for England, our FGC adult research and practice network group, and by directly approaching organisations that had indicated an interest in setting up an adult FGC service or had already set up one. Despite adopting an active dissemination strategy, we received a low response rate (n=7), suggesting a low level of current adult FGC activity in the UK. Following consultation with the study’s strategic advisory group, the survey was reopened for two months (May and June) to confirm that the low response rate was due to the scarcity of services offering FGC. Alongside reinstating our survey, a simple poll using Online Surveys was created to allow organisations an opportunity to share their longer-term plans regarding FGC. This was primarily conducted to gather responses from a wider pool of organisations with a potential interest in FGC to better gauge whether our low response rate was indicative of the actual level of FGC activity across the UK. The poll was circulated via the Chief Social Worker (adults), the Principal Social Workers’ Network for England, and equivalent networks in Scotland and Wales. We were informed that although there had been an initiative to trial FGCs for adults by the integrated Health and Social Care Board in Northern Ireland, no actual conferences were offered and the initiative was no longer active. Responses to this poll elicited 16 responses, all but one indicating no plans to set up an FGC service in the near future, confirming our capture of the limited extent of current FGC activity in the UK.

Data was descriptively analysed through the analytical functions of the Online Survey. For closed-ended questions, frequency tables and charts were used to determine the number of responses to each option. Open-ended questions and/or responses to free text boxes were subjected to thematic analysis, mapping them against themes generated through the literature review whilst also identifying outliers. Our analysis helped further refine our initial characterisation of the FGC model(s) generated from the literature review.

**Stakeholder interviews**

To gain a more contextualised and holistic understanding in relation to how current FGC services were operating, we interviewed a purposive sample of stakeholders connected to the services from whom we had received survey responses. Survey respondents who agreed to be contacted for an interview were invited to participate in our stakeholder interviews, and additional participants were sought using a snowballing approach within local service networks. A total of 11 interviews were conducted between July and August 2023 with a cross-section of Team Managers, FGC coordinators, referring social workers, and people with lived experience (n=3). Upon sending an initial email invite to potential participants, information sheets and consent forms were sent, providing an overview of the study and detailing their rights as participants. Upon receiving a positive response, an interview date for a virtual interview was set up.

Our survey themes formed the basis of our interview topic guides, which were drafted in collaboration with peer researchers. These were then refined with input from our Lived Experience Advisory Panel (LEAP), a group of people with lived experiences of either being the person at the centre of the conference or being a network member. This was instrumental in ensuring that relevant questions reflective of a service user perspective were included and that the language used was simple and appropriate. The development of the interview topic guide for service users was led by peer researchers, with input from a wider research team. Service-user interviews were undertaken by peer researchers, as they were deemed more effective in establishing rapport and facilitating an open and honest conversation. Peer researchers also actively contributed to our topic guide developed for professionals by providing feedback on the inclusion and/or deletion of specific questions. In addition, one peer researcher participated in coding and analysis of the interview data.

Interviews were analysed using a hybrid approach to thematic analysis, using the overarching framework identified in the literature and a national survey. To arrive at codes related to our themes, we used inductive thematic analysis, which provided us with rich explanations, corroborations, and/or variations among our datasets. In doing so, our interview data supported the successful integration of findings at the theoretical level ( Proudfoot, 2022). Upon completion of our analysis, the findings were sense-checked with the LEAP, who provided corroboration that these fit well with their own experience with an FGC.

**Deliberative forum**

Deliberative methods are best viewed as an approach to research rather than as a method in itself that aims to involve people in decision-making in a meaningful way ( Rawls, 1997) while also providing an opportunity for participants to discover more about a topic and discuss with other participants before presenting their views. Employing this approach was particularly helpful, as it helped us arrive at a broad agreement (or disagreement) among all participants ( Burchardt, 2012) especially on contentious issues that were identified through the survey and/or interviews. Particularly, we were able to gain ‘informed views’ ( Carson, 2006) by providing prior evidence from our literature review, survey and interviews in a simple and digestible manner ( Barnes, 2008) thereby gaining maximum benefit from the deliberation process.

The invitation to the Deliberative Forum was extended to all our survey and interview participants, and via them invitations were cascaded to others with involvement in relation to FGCs in their localities. We also extended invitations to our LEAP members, other statutory and non-statutory organisations interested and/or involved with adult FGC, and academics researching FGC or related approaches. From our synthesis of evidence from the literature, the national survey, and stakeholder interviews, we provided participants with a document in advance that summarised where we thought we already had clarity around the model, process, and outcomes, and what stood out as the main unanswered questions where evidence seemed to be lacking, unclear, or potentially contradictory. 

Participants (n=36) were divided into four subgroups, with each group focusing on a specific set of questions. Each group comprised people with practical, academic, and lived experiences. To effectively capture discussions, each group was assigned a facilitator and note taker, who then fed back a summary of their deliberations to the wider group for further reflection and discussion. Summary feedback and subsequent discussions were digitally recorded to assist with our analysis. In addition, arising out of the small group discussions and wider group responses, there emerged some additional points relevant to the FGC process and outcomes on which there was broad agreement, which were also captured. Findings from the Forum assisted in filling notable gaps, validating our previous findings, and highlighting variations in practice models.

### Characterising the model – findings from the literature and the practice field

Although emerging practice model(s) of Family and Group Conferencing for adults may differ significantly from what has become an established practice with children and families, there is still substantial common ground. In both adults and childrens contexts, FGC is an inclusive process in which an individual and their social and family networks come together to make plans in relation to the care and support of a potentially vulnerable individual. Facilitated by an independent coordinator, FGCs follow a staged approach to planning and decision making. Although subject to some degree of flexibility, the basic outline of FGC in an adult context may be characterised as follows:

‘During phase one, the preparation phase, the coordinator helps the central person to explore [their] social network. The person decides whom to invite the conference. Subsequently, the coordinator contacts the selected members of the social network and has preparatory talks with each one of them. The coordinator also helps the central person to formulate the main question, picks a time and place, and helps decide whether or not and which social and/or medical professionals to invite. Phase two, the information phase, is the start of the meeting, during which professionals can provide the information needed to help answer the person’s main question. The participants can ask for clarifications and more elaborate explanations. In the third phase, the private time, the person, and [their] social network deliberate together; the professionals and the coordinators are not present. The network members formulate a plan and decide how to evaluate it. During the fourth phase, the participants present their plan to the coordinator and the professionals involved. The coordinator helps finalize the plan and make it as concrete as possible. ( Metze *et al.*, 2015 p.168)

As will be discussed in more detail later, there is less clarity or consistency in terms of the final stage of the process in which the plan is implemented, reviewed, and potentially modified to take into account changing circumstances and people’s experience of how it is working. While Metze and colleagues (2015) proposed that the fifth phase (carrying out, evaluating, and adjusting the plan) is ‘entirely up to the central person and their network’ (ibid.), it was reported to us that it is usual practice in UK FGC services for the coordinator to oversee some form of the review process in conjunction with the central person, network members, and relevant professionals.

The typical context and purpose of an adult’s FGC can be subtly different from those of a children ’s FGCs. While the purpose of the former is often for a family to develop a plan that satisfactorily addresses professional concerns regarding the safety or welfare of a young person, the purpose of the latter may be seen as more explicitly emancipatory: it is about enabling an adult to have control over their life in conjunction with the people that matter to them. It may be seen ‘not as a source of assistance, not as an intervention or help, but a decision-making process that makes citizens active’ (Van Pagée, 2007, cited in Jenkins, 2010 p.2).

**Values and principles underpinning Adult FGC**

A consistent theme from the stakeholder interviews was that, while flexibility and creativity in practice were to be encouraged (in itself a value statement), it was crucial to the integrity of the process that it remained true to the core values and principles of Family and Group Conferencing, and this is echoed in the literature ( Merkel-Holguin, 2004). However, there is currently no clear and agreed statement of what these values are, and these may be more implicit than explicit. Therefore, in characterising FGC practice for adults, it will be important to tease out the underpinning values and principles.

First, it may be seen as a privileging of a relational perspective over an individualistic one:

The general vision of human beings which underlies FGC method [is] the vision that people are social beings and dependent on each other for their wellbeing and happiness, especially when they feel vulnerable’ ( Metze *et al.*, 2015 p. 169)

Sustainable improvements for an individual are seen as being more likely to be achieved if family and friends are directly involved in identifying and finding solutions to current difficulties ( De Jong *et al.*, 2018; Ramon, 2021). Within the context of mental health, interrelationships and connections may be crucial to the recovery process ( De Jong *et al.*, 2014; Tew *et al.*, 2012; Topor *et al.*, 2006).

Perhaps reflecting more potentially coercive scenarios where the professional system may be poised to use statutory powers to intervene, children’s FGC literature frequently emphasizes a rights-based perspective – a human right to a family life that is not overridden by the state wherever possible ( Pennell *et al.*, 2011). By contrast, in the context of adult social care in the UK, similar statutory powers do not exist, leading to a less potentially coercive ethos (although there may still be professional concerns), and such an explicit rights-based perspective is much less a feature of adults’ literature. Similarly, from our interviewees, there was less of an explicit view that the option of an FGC should be a human right, although an implicit right to self-determination wherever possible was seen to underpin FGC practices. Instead, they saw FGC as primarily being about organizing care and support by leveraging individual, network, and community resources and in a way most relevant to the central person.

FGC is seen as an ‘inclusive and democratic approach’ in which all viewpoints are valued ( Merkel-Holguin, 2004; Tew *et al.*, 2017), and there is a clear consensus both from the literature and from the interviews that a defining value of FGC is that the process must be family and network led, focusing on what matters to the individual at the centre and their family/network members ( Górska *et al.*, 2016). Being ‘family led’ suggests FGC can empower families and wider networks to support each other to find solutions to the problems they face. In short, it gives families ownership of decisions made ( Hobbs & Alzoni, 2013). Jenkins emphasizes a focus on the strengths, capabilities, and leadership of citizens and family networks in decision-making’ ( 2010, p.1).

The practice experience that was shared in the stakeholder interviews suggested that putting this value into practice was not always straightforward; for example, when a particular family or network member had their ‘own agenda, ’ which might not be aligned with the needs and wishes of the person at the centre of the process:

*“If there’s somebody perhaps who very clearly has a particular vested interest or position that they are very clearly coming from and they are not putting the wishes and needs of the person at the centre of it, then I guess [an FGC] might be difficult”* (interviewee 3- social worker)

There could also be issues where members of the professional system were uncomfortable about the coordinator stepping back and giving power to the person and their network to make their plan without professional oversight. Similarly, there could be concerns from individuals and/or members of their network that they should not be left alone to make plans or decisions without professional assistance. Some interviewees suggested that this could be due to the legacy of previous experiences of services that had ‘done to’ or ‘done for’ people, taking away their confidence that they could do things for themselves. However, in other instances, this might be due to real concerns that dominant or potentially abusive members of the network might exert undue influence over the process and outcome. It was felt that coordinators should have the flexibility to use their judgement to adapt structure and format to maximise people’s abilities to work together, resolve conflicts, and achieve outcomes that they could own.

Central to the model is valuing the specific knowledge and expertise that family and network members have in relation to their situation:

Who knows what is best for the people, if not the people themselves? This question, which reflects the core principles of democracy, also is central to the practice of family group conferencing’ ( Merkel-Holguin, 2004 pg. 155; see also Hardy, 2017).

In contrast to what may be usual in many professionally led decision-making processes, it is this knowledge and expertise that is seen to be paramount in shaping the decisions that are made, although professional views and assessments are also shared, discussed, and valued as part of the overall process.

Equally central to the model is the principle that the coordinator should be independent ( Górska *et al.*, 2016) and not act as either a representative of the wider social care or health system or as a provider of (or gatekeeper to) other services. There was consensus within the interview sample that (a) the coordinator should be separate from other professionals (such as social workers), and (b) their role within the FGC process should be limited to coordination with no input into decision-making. Their role should not be to offer expertise, influence outcomes, or make suggestions as to what should go into the plan. Instead, their role is simply to facilitate a process whereby the person and their network can devise their own plan.

“ *The independence is vital and particularly sometimes if you have disagreement with a professional who’s holding the case. It brings people together with an independent person facilitating and a neutral venue in which to hold the meeting, two really important things there*. (Interviewee 7, FGC team manager)

While acknowledging this as a core principle, informants from the practice field shared how it was not always easy to uphold this in practice – for example, where services were being cut back, and it might seem pragmatic for the coordinator to take on other roles or tasks in order to fill gaps in provision.

Although ultimately all decisions are made by the central person and their network, informants from the field of practice stressed how the independence of the coordinators also allows them to take on a challenging or even a ‘devil’s advocate’ role by encouraging individuals in thinking through some of their choices or decisions. A common example cited was gently challenging, where a person might be reluctant to invite someone who could be potentially important in relation to their support – perhaps due to feelings of shame or not wishing to burden people.

There can be a strong alignment of values between FGC and the wider field of restorative practice; however, themes of reconciliation and dealing with past harm may not be relevant in all FGCs. The focus on making a plan imbues FGC with an inherently forward focus, and there is a commitment to avoid destructive processes of blaming ( Fisher *et al.*, 2018). A restorative approach provides space for openness in which feelings of shame can be shared ( De Jong & Schout, 2013) and in which participants can agree to acknowledge past experiences of hurt or harm and share and listen to each other’s feelings in relation to these, so as to be able to come together and resolve their current issues.

Although there is general agreement across the literature and the practice field that FGC services should be culturally appropriate and accessible to all communities, there is a tendency in the literature to take the cultural adequacy of FGC for granted and not question how successful FGC is in challenging paradigms and decolonizing social work ( Nygard & Saus, 2019). Because FGC was originally developed for Māori families, it is not necessarily culturally appropriate for other contexts. These authors argue that implementing FGC in new communities requires a strong foundation in the local cultural context. Based on their interviews with practitioners, Barn and Das (2016) proposed that, wherever possible, people should be offered the option of a coordinator who is matched on the basis of a shared ethnic, cultural, and/or religious background. Overall, it is suggested that the inherent informality and non-institutional nature of FGC practices can facilitate central persons and their networks in dealing with problems in a way that is consistent with their own culture and lifestyle ( Meijer *et al.*, 2019).

A core value of the FGC model is that it seeks to determine the strengths of individuals, their families, and social networks ( Hardy, 2017). Similar to other strength-based and (potentially) preventative approaches in social care ( Tew *et al.*, 2023a), FGC is predicated on the changed relationship between the state and citizens in which welfare practices are no longer ‘done to’ disadvantaged or vulnerable people, and they are instead invited to be active agents in determining what would be a good life for them and how to achieve this ( Blundell *et al.*, 2021; De Jong *et al.*, 2018; Metze *et al.*, 2015). Ramon (2021) highlighted that the recovery approach to mental health is also a relevant practice framework within which FGCs can operate, as this prioritizes reclaiming a satisfying and meaningful life that can, in turn, lead to specific mental health difficulties remitting or being better managed.

However, beneath this, there is a political tension as to whether FGC is seen as an emancipatory practice that enables citizens to take control over their lives by making relevant decisions to them ( Blundell *et al.*, 2021; Frost *et al.*, 2014), or whether it is seen as fitting with the neoliberal agenda of rolling-back state support ( Bredewold & Tokens, 2021; see also Jackson & Morris, 1999). Within international contexts such as Holland, the development of adult FGC has been seen as linked to a political movement in which citizens have increasingly been expected to be ‘self-reliant’ and assume responsibility for their own care needs as well as for those others close by in their communities ( Bredewold *et al.*, 2020). Within the UK context, some practitioners reported having to steer a path in their conversations with families where they acknowledged a wider landscape in which social care or health services were being cut back but did not situate FGC as a mechanism whereby such cuts could be achieved.

More broadly, stakeholders located FGC within a wider context of implementing strengths-based, person-centred or recovery-oriented approaches, with a focus on the (potential) capabilities of individual, network and community to enable the central person to live what would be, for them, their ‘best life.’

*“The potential for working with a person’s strengths and switching completely from that care approach where we would advise services based on an assessment that looked at what the person cannot do. I really liked the idea of what a person can do”* (Interviewee 7, FGC team manager).

Within mental health settings, FGC is perceived as a unique service that offers a value-based alternative to the medical model, which can often be dominant, particularly within health settings.

*“I mean in theory it’s perfect, it’s amazing. It was put in place to enhance our strength-based practice and when it works, it works really well”* (Interviewee 1, FGC team manager).

**Role, training, and professionalism of FGC coordinators**

The role of the FGC coordinator is seen as crucial in all FGCs, particularly in the preparation phase, and is crucial in ensuring that relational experiences in FGC are supportive. There is consensus across the literature and practice informants that the role requires considerable skill and sensitivity, particularly in more complex situations where there is conflict in the network, or where the central person may need a lot of support in building up their confidence and ability to articulate their needs and preferences. Where there may be concerns about abuse or safeguarding, it may be seen as important that the coordinator has ‘an in-depth knowledge about family dynamics and the impact of intimidatory dynamics on processes of participation’ ( Connolly, 2006, p. 350).

However, within the literature, there is quite a divergence of view as to whether the role is best undertaken by a citizen with relatively little formal training – ‘a gifted citizen with natural abilities to mobilise groups’ ( Schout & De Jong, 2017 p. 1198), or whether it should be a much more professionalised role, perhaps undertaken by someone from a social work or social care practitioner background. Proponents of the former approach, the model developed in Holland, argue that this is the best way to ensure that the coordinator is seen as a ‘neutral fellow citizen’ who is completely independent of state services (ibid). A contrasting view is presented by Blundell and colleagues who suggest that the role of the FGC coordinator (in this case working with adults experiencing elder abuse) involves the ‘ability to assess risk and signs of safety, negotiate directly and unambiguously, challenge effectively while understanding and respecting network cultures and processes, [all of which] requires sophisticated and ethical practice skills and processes’ ( Blundell *et al.*, 2021 p.313).

Currently, there is no specification and regulation on training for adult coordinators in the UK, and the norms within the field are either that coordination is undertaken by practitioners who have trained in children’s FGC work and then take on work with adults, or that bespoke adult-focused training is offered, either by an independent training provider or by more established FGC services. Interviews with stakeholders indicated that initial training may be as few as three to four days, and this is then supplemented by shadowing opportunities, co-working, and service-based continuous professional development. Professional supervision usually comprises a combination of peer group supervision and supervision by an FGC service manager. Hobbs and Alonzi (2013) raise some concern about the wide variety of training and experience of FGC coordinators when working with ‘at risk’ adults. They contend that further work is required to provide training and accreditation for FGC coordinators in the adult context to ensure standards and consistency in practice.

From the practice survey and stakeholder interviews, we established that there are currently a variety of ways in which coordinators have been employed in the UK, typically either separately from or in combination with statutory responses. Perhaps offering the greatest independence to coordinators is the model in which coordinators are either independent contractors or employed by a dedicated voluntary sector provider, and their services are purchased on a case-by-case basis by mainstream services. When coordinators are independent, they are expected to be responsible for their own training and continuous professional development. Some adult FGC services are nested in-house in local government or health service organizations – managed at arms length from community or hospital teams and therefore able to maintain an ethos where they are clearly seen as being separate and independent. Where levels of adult FGC activity may be limited, coordinators may be based on teams primarily located alongside children’s FGC services or as members of an adult social care team. This latter configuration offers the advantage of greater visibility and connection with wider social care activity, but may make it harder for coordinators to feel and be seen as fully independent.

***For whom may an FGC approach be suitable?***

**Figure 3.**
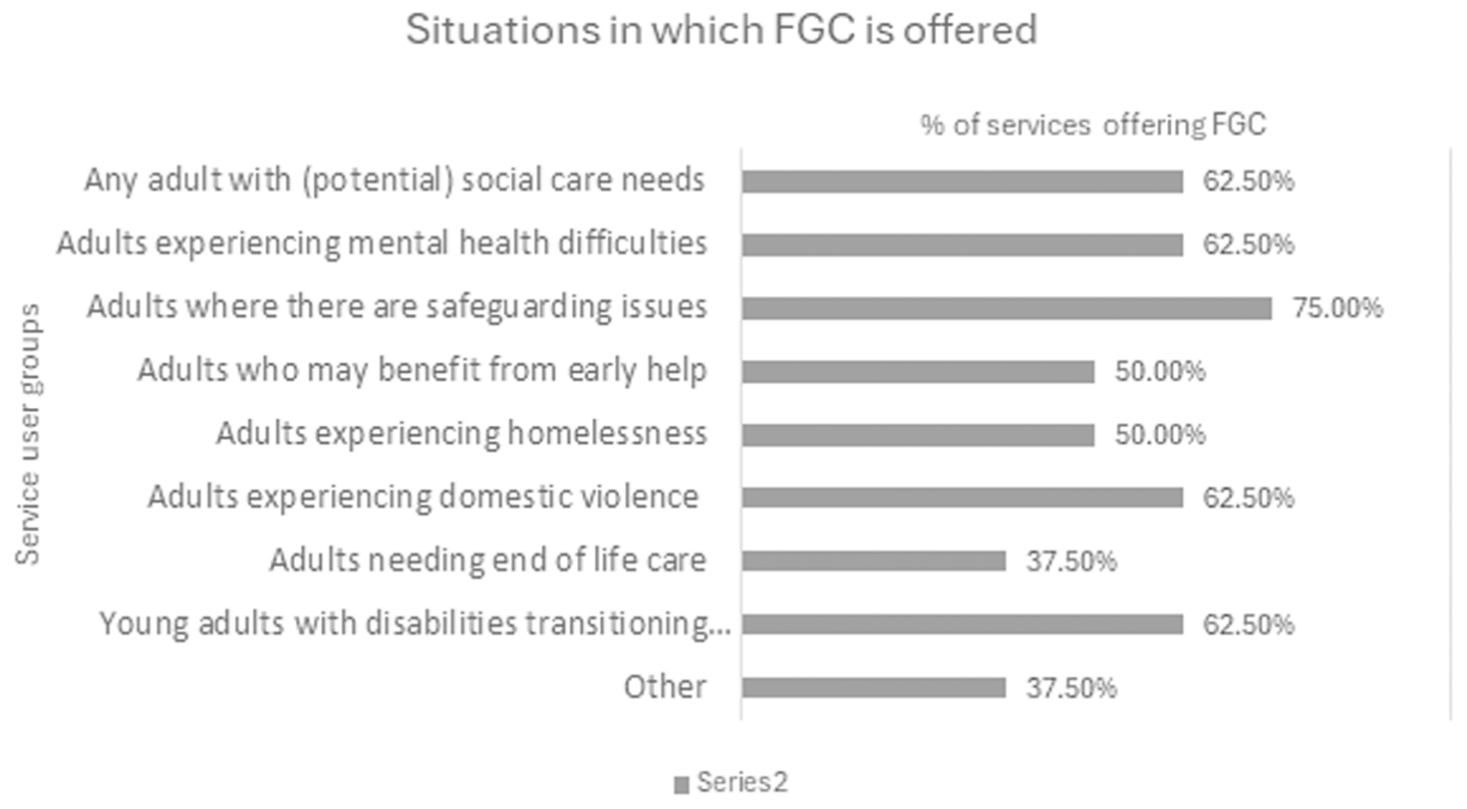
Circumstances in which people may be offered FGC services in the UK – practice survey.

Both the literature and the practice survey indicated the wide applicability of the FGC approach across adult social care and mental health sectors, with relevance across the board for people with learning or physical disabilities, older people, and people with mental health difficulties (see Figure 3).

Fisher *et al.* (2018) summarise the range of contexts in which adult FGCs may be used in the UK:

•   Where there is a breakdown of (or need to review) current care and support arrangements, a challenge in relation to mental or physical health or functioning, social isolation, homelessness, or a need to plan end-of-life care.

•   Where service contexts are changing, for example, at the point of hospital discharge or transition from support from children’s services.

•   There are professional concerns about safeguarding in relation to personal safety (for example, for people with dementia), abuse or coercive control, neglect and self-neglect, substance misuse, or hoarding.

While it can be helpful in setting up an FGC if there are already respectful and trusting relationships between people and support services ( Ramon, 2021) it may also have particular value in situations where this is in itself an issue. For example, in Holland, FGCs have been offered to people with multiple problems who would ideally need care, but do not access it or ask for help ( De Jong & Schout, 2011). Specifically, in relation to mental health, conferences have been offered in the contexts of voluntary help, assertive outreach, and the possibility of involuntary admissions (ibid.) However, there is an argument that offering FGCs in more coercive contexts may not necessarily provide the conditions for a process that is citizen-led and empowering.

There were divided opinions reported in the literature as to whether FGC could be appropriate for use in crisis situations:

‘Often professional help is needed to address the crisis, but to make a plan for what needs to be done when the crisis is solved and how to prevent the next crisis happening, a family group conference can be a good approach’ ( De Jong & Schout, 2011 p.70).

One possible model that was suggested was to convene an initial professionally led crisis meeting to agree with a short-term plan to hold the situation and make arrangements for immediate safety and support. This would then create a sufficiently safe space in which more thorough preparatory work could be undertaken for a Conference, including, where relevant, looking to widen the network so that a more sustainable system of support might be achieved.

There may be circumstances in which the central person may lack the capacity to make an informed decision regarding participation in the FGC or to participate in the process in a meaningful way, for example, due to more advanced dementia ( Górska *et al.*, 2016). Similar concerns were encountered in situations where participating in a Conference was likely to aggravate psychotic problems ( De Jong *et al.*, 2014). In principle, it was seen as possible to proceed with an FGC process in which only network members participated in the actual Conference but with the interests and wishes of the central person still being represented within the conference discussions, perhaps through the involvement of an advocate. There may be other scenarios in which the central person elects not to be present at the Conference itself but is happy for the process to proceed with their wishes being represented by other means, perhaps via a recorded statement or the briefing of another person to speak on their behalf. 

Metze (2007) cautioned that FGCs may not always be appropriate in situations of domestic violence, reporting both an instance where the FGC resulted in an effective plan with good results and one where there was minimal support provided and no evidence of change for the better. However, Tapper (2010) reports more positive results with a nearly 50% reduction in the number of domestic violence crimes reported since have an FGC and a 73% drop in approaching the police 6 months after the FGC. There may be possibilities for restorative change, but also dangers and challenges, if the perpetrator of the violence is allowed into the room as part of the process ( Sen *et al.*, 2018). To mitigate these, interviews highlighted the option of conducting two separate conferences or holding hybrid conferences to ensure the safety of individuals.

While Blundell and colleagues suggest that FGC has the potential to put an older person experiencing abuse ‘at the centre of the intervention and focus on their context and relationship’ ( 2021 p. 311), it may not appropriate in all cases because of the intersection of contributing risk factors, ‘including intrafamilial dynamics, mental health, intergenerational transmission of violence, the impact of cognitive impairment and dependency’ ( Blundell *et al.*, 2021 p. 314). Nevertheless, they argue that FGC can be successful in working with elder abuse if sufficient resources are put into preparation and implementation of the agreed plan.

Based on their experience of offering FGCs in situations where there were safeguarding concerns, Daybreak (a voluntary organization that has offered adult FGCs) proposed that the decision about whether to include a perpetrator of abuse in the FGC should depend on:

- Whether the victim of the abuse has an ongoing relationship with the perpetrator and intends to continue.
- Whether the victim wishes for the perpetrator to be included in the process
- If the perpetrator acknowledges the abuse and wants to change their own behaviour.
- Any risks identified to other participants. This will include the victim, other family, and professional participants. (2010 p.10)

Ground rules may need to be established, with the central person having the right to withdraw from the meeting if they feel that they are unsafe or intimidated, or network members may be asked to withdraw if they are acting inappropriately. Where a central person may lack capacity, a meeting of just network members may be a viable way forward to plan for their safety.

In situations where people did not feel that they had the basis of a viable network, there were examples in the literature from the Holland of people feeling pressured to invite neighbours or others to be part of their support network ( Bredewold & Tokens, 2021). This could be humiliating and undermine rather than support people’s weak social networks. ‘Forcing people to ask relative strangers (may) merely add shame to their loneliness’ (ibid. p.2185). One interviewee described similar scenarios, in which they were unable to proceed following a referral because they had no success in identifying people who might be potential members of a network of support:

*“Where are you going to find a network of support?’ Unfortunately, we have such situations too,”* (Interviewee 5- FGC team manager)

Though we gained limited data on issues around cultural appropriateness, some participants highlighted that for some individuals, FGC may not fit well with their accustomed ways of doing things, perhaps due to issues around stigma and family shame associated with external ‘help seeking’ behaviours. In some instances, ‘ *people with certain religions might say … we could sort it out for ourselves, we don’t want somebody coming in”* (Interviewee 2, social care practitioner). In some instances, FGC may feel inappropriate due to strongly embedded age- or gender-based hierarchies in which women might not feel able to speak out openly in front of men, or members of a younger generation might not feel able to question the judgement of their elders.

***Descriptions of format and process***

Consistent across our interviewees and survey responses, the FGC format is seen to consist of a preparation phase, conference phase, and review or follow-up phase. Within this framework, stakeholders emphasized that effective engagement depended on coordinators being able to be flexible with the format of the overall process to suit people’s particular circumstances and preferences.

**Preparation phase**

From both interviewees and the literature, it was clear that the preparation phase is crucial to the success (or otherwise) of the conferencing process. Broadly, the central role of the FGC coordinator is to create the conditions and support for a process wherein the central person and other participants are willing to meet ‘on a level’ with each other and be able to behonest and open about their concerns, aspirations, and preferences. If appropriate preparation and support to meet and communicate respectfully does not exist, the FGC experience may become humiliating rather than helpful ( Blundell *et al.*, 2021).

Although the coordinator will benefit from some information through the referral process, their first task is to establish contact with the central person and gain an initial understanding of their situation, their concerns and aspirations, and the people around them who are currently (or potentially) part of their support network. Sometimes, a central person may be reticent about inviting certain people (perhaps due to feelings of shame or embarrassment), and the coordinator may have a role in gently challenging this and encouraging them to invite all the people who matter to them. In most services, the central person appears to be at the centre of all decisions made regarding which network members and professionals should participate. However, such decisions could partly be influenced by the professional judgement of the co-ordinator regarding ‘important and relevant’ persons close to the central person and/or by any safeguarding issues that may have arisen – although this would be done in a way that still left final decision-making power:

*“I suppose part of my role is to kind of encourage who would be the best people to be at the Conference, but in a way that I’m not kind of dictating’* (Interviewee 4- FGC coordinator)

In addition, the coordinator establishes contact with relevant professionals and services to elicit their input and, if possible, their willingness and availability to take part in the first part of the Conference – something that may not always be straightforward, particularly where other services are unfamiliar with the FGC process:

*“The other boundary I suppose to that is whether physical health services see this is as a bit of a, ‘What is this? This isn’t part of my role. Why am I being invited to this?’* (Interviewee 4: FGC coordinator).

There may be a need for the central person to have an advocate, a role that may be provided by someone from an advocacy service or, quite commonly, by another FGC coordinator that would bring the added benefit of greater familiarity with the FGC process. There was some variation between services in the degree to which independent advocacy was offered, partly reflecting differences in typical clientele (and their levels of capacity), but also reflecting whether the preparation process itself (such as the formulation of a personal recovery plan) was usually seen as sufficient to ensure that the central person would have a voice in the decision-making process. However, any blurring of the coordinator / advocate role may perhaps be seen as undermining the perceived independence of the coordinator – and the potential to manage this could depend on the co-ordinator’s own training and experience in this regard.

Although the interviews highlighted some differences in the precise way it would be put together, there was general agreement that a working document would be circulated to everyone before the Conference. This document would include all relevant information and concerns to ensure that there are no surprise disclosures of agendas on the day of the Conference, and it would be written in a way that foregrounded the aspirations and preferences of the central person. 

**Conference**

Our interviews and surveys highlight a common set of frameworks and practices within the Conference phase. However, services appeared to have adopted significantly different approaches to how these were operationalised. The choice of venue was considered important, and there was consensus that health or care service settings were generally inappropriate. Neutral venues, such as libraries or community centres, were often used to conduct the conference; however, in some other services, people’s homes were also considered as an option. Where homes were used, this would be where individuals preferred it or if they had mobility issues.

*“We’ve got a selection of preferred venues around the borough that I use. And we would suggest to an adult, well we’ve got this, we’ve got that. But sometimes they say, well actually there’s this little placse round the corner that I go every Wednesday afternoon so we may approach there,”* (Interviewee 7- FGC team lead).

In addition, flexibility or variation appeared in the format of the Conference. While in general, planning and decision-making took place within a single inclusive Conference, there could be alternatives in terms of two or more mini-conferences, and online or hybrid options, in order to better include different network members (including those who may be geographically dispersed), or in situations where there may be issues around abuse or safeguarding.

*“So obviously [a hybrid option is] not ideal because it would be helpful to have everyone in the room, but equally we’d rather have that than no conference at all,”* (Interview 4- FGC coordinator)

The Conference itself may be seen as comprising four stages as outlined below:

(i) Getting together.
Although not universal as a practice, many informants strongly favoured an approach in which the participants came together in an informal and equalizing way before the main business was conducted. This would usually involve the sharing of food, thereby setting a tone that is very different from conventional professionally led decision-making processes.

(ii) Introductory Discussion
  The agenda for the Conference will have been agreed beforehand with the central person and network members, and will foreground their concerns and aspirations. However, it may also be necessary to reflect on the specific concerns raised by the professionals involved. This is read out at the beginning, followed by an agreement on any relevant ground rules, for example, to ensure that all voices are heard.
The first part of the conference provides space for potentially extensive discussion with invited practitioners. This is facilitated by the coordinator and places the person and their network very much ‘in the driving seat’ in focusing on what matters to them, rather than following an agenda dictated by professionals ( Tew *et al.*, 2017). Ideally, it can provide for a shared ‘learning platform’ in which both professional and network participants can move beyond initial viewpoints and preconceptions and discover new and better ways of understanding what may be going on (and why) ( De Jong & Schout, 2013).

Feedback from interviewees indicated that the process could be very different from that of a professionally-led meeting or a Ward round ( Tew *et al.*, 2017), with both network participants and practitioners reporting (and appreciating) conversations that felt genuinely dialogical. This suggests strong parallels with the process of network meetings undertaken as part of the Open Dialogue approach in mental health ( Seikkula, 2011). A key underpinning principle common to both FGC and Open Dialogue is the importance of practitioners being willing and able to live with uncertainty while issues are explored, so that there is space for solutions to emerge from the central person and their network, rather than these being proposed (or imposed) by professionals ( Seikkula *et al.*, 2003; Tew *et al.*, 2025b).

(iii) Private time
This introductory discussion provides a prelude for private time, in which it is expected that both professionals and the coordinator withdraw to allow the person and their network to have as long as they need to develop their plan for support, enablement, or recovery. Advocates would usually stay to support the person in articulating their voice during private time. The coordinator will remain on hand and may be invited to help with specific aspects of the process (but not advise on what they might decide) and may just check in at regular intervals to see how their discussion is progressing.
While most interview participants shared that private time was to occur exclusively in the absence of professionals, we noted a few instances where the coordinator was asked to stay back to support the individual in articulating their thoughts and/or assisting the network in articulating their plan.
  “ *I know a traditional FGC private time is family time. However, I have attended private time probably a couple of times. Not because I’ve wanted to, it’s because the family just was like a rabbit in headlights when we said, ‘Right okay, you’re going to make the plan.’ And although I explained to them why we remove ourselves from family time and the benefits of that, some families are just like, ‘No’.”* (Interview 6: FGC coordinator)
(iv) Feeding back and finalising the plan
Once the central person and network members have finished their deliberations in private time, they share their ideas with the coordinator, who can (if needed) help in firming this up as their agreed plan ( Metze *et al.*, 2015). Where plans involve the provision of services or the need to address issues relating to safeguarding, relevant professionals may be contacted to explore the feasibility of offering their support. In instances where professionals are absent during the Conference, plans may be confirmed only after input from other professionals. Where there may be reservations in fully supporting the plan, the coordinator may negotiate with professionals on behalf of the central person or explore alternative support offers. The feedback session may also include a discussion on how they will be able to evaluate how well the plan is working.

**Review/Follow up**

Our participants shared the perspective that some form of review and/or follow-up is important in determining the progress made with the plan. While FGC reviews may be seen as a necessary part of the process to sustain resilience and maintain momentum ( Fisher *et al.*, 2018), we noted variations in the purpose of offering a review, the format of the review process, the number of reviews offered, and the timing of these reviews. Such variation could be between different services and also within services, depending on the preference of the individual, but equally on the contents of the plan. Similarly, we noted variations in who was invited to the review, the setting for the review (choice of venue or online), and the timing of offering the review.

“ *We always offer a review, sometimes it’s face to face, sometimes it’s just on the phone, just to check how everything’s going, obviously it depends on what the action plans are*” (Interview 1- FGC team lead).

In most services, a review is offered to determine the immediate progress with plans, usually four to six weeks after the Conference. In some services, reviews were also used to address other issues that may have arisen, and perhaps, two or three further reviews could be offered to support and adjust the implementation of the plan over a longer timescale. Some services stipulate a maximum number of reviews. In some instances, reviews can be used to celebrate success with all involved. There was some lack of clarity as to what extent (and for how long) a coordinator could (or should) remain involved to ensure that what had been agreed was actually being delivered, either by services and/or by network members. In some instances, such follow-through would be undertaken by the referring practitioner, but, particularly when services were stretched, there could be a danger that there would be no one who could provide this sort of ongoing support, with potential implications for the sustainability of positive outcomes.

**Confirmations and clarifications from the Deliberative Forum**

Prior to the forum, we circulated a summary of what we thought we understood based on the findings discussed above, and what remained as key unanswered questions or areas of contention. There was broad agreement with much of our preliminary characterisation of the model, including its outline structure, values and principles, the role and independence of the coordinator, and the potential applicability of the model across all adult service user groups. 

There were three key questions relating to our preliminary characterisation of the model that we brought to the forum. As outlined in the previous sections, there is a limited understanding of the feasibility of offering FGCs in specific situations, such as those in a crisis or the conditions necessary when offered to those with diminished capacity. Similarly, we considered that assumptions about the cultural appropriateness of FGCs needed to be explored more rigorously to gain an understanding of whether current model (s) are able to adapt the process to accommodate the social and cultural requirements of the central person and their networks. While there was consistency within the literature and interviews regarding the flexibility of FGCs, there was a lack of clarity on the aspects and extent to which flexibility was acceptable. These questions were presented in the Forum, and the responses that emerged from the deliberations are summarized in Table 2.

**Table 2.** Summary of deliberations from the Deliberative Forum.

Question	Responses
When should people be offered an FGC service? For example, can an FGC be offered • in a crisis situation • where there is limited or no network • where a person has diminished capacity • where there are significant relationship issues?	• In general, FGCs can be considered as an option in any situation. • FGCs may be suitable in crisis situations – typically not in ‘acute’ crisis situations where immediate safeguarding issues may need to be resolved first, but in more ‘chronic’ situations where current support arrangements are breaking down or not working. • In a crisis situation, FGCs can bring agencies and network members together and provide a constructive structure within which to manage emotions. • FGCs can be offered where there is limited and/or disengaged networks. In such situations, coordinators need to be creative in thinking of ways to build a network – although accepting of the possibility that no-one may engage, and a Conference may not take place. • Where a central person may lack mental capacity, an advocate can provide support and act on their behalf. • If the central person is not able or does not want to be involved, those offering care can become the focus of the FGC instead. • Whilst FGCs can be offered in situations where there are significant relationship issues, these need to be handled carefully in preparing for the Conference.
Are FGCs appropriate for people from all cultures and social backgrounds? • In engaging with traditionally patriarchal families • Where there are overriding concerns of social shame	• Whilst an FGC can be offered and can work for people from all cultural groups, there can be some reluctance to engage among some communities – for example, people from Pakistani and Bangladeshi communities. This reluctance can stem from cultural expectations of showing respect and deferring to senior family members. • Across cultures and ethnic groups, people may want to show ‘their best side’ rather than their weaknesses and not want to expose the family to shame. This can affect engagement. • Ethnic or cultural matching of coordinators should be offered as an option – but may not always be what people want. • When thinking about shame, it is important to consider wider societal factors (such as racism, stigma and social exclusion) as well as specific cultural norms - it is not always cultural norms that may be leading to shame.
How flexible can the overall ‘package’ of FGC be to take account of culture, safeguarding or capacity issues etc? Are there any circumstances in which co-ordinator and/or practitioners should stay for private time if invited to do so?	• FGCs can be flexible to take into account the needs and background of the person and their network • The venue, location, hybrid options, equipment, having enough time – all these elements can be flexible. • Flexibility means holding on to core values and principles but having the perspective ‘to see where it goes’ instead of having ‘a fixed package’ of things to do. It is about enabling dignity, respect, autonomy, collaboration, and having equal power. • In cases of safeguarding, there may be less flexibility as the FGC may take place within the constraints of wider structures, operational policies and practices. There were no conclusions reached as to whether and under what circumstances a co-ordinator and/or practitioners should stay for (parts of) private time if invited to do so.

While FGCs may be offered in all situations, including to those experiencing a crisis, they may not always be appropriate as a first response in an acute crisis, especially where there may be safeguarding concerns that may take precedence, but it can be a viable option once immediate problems have been addressed. However, in any type of crisis, FGCs can provide an opportunity for services and networks to come together to manage a situation in a more collaborative manner. There was agreement that there needed to be flexibility and creativity where the central person may lack some degree of capacity in relation to particular aspects of decision-making. It may be useful to employ an advocate to engage with the central person to establish their wishes and preferences prior to the Conference and find the best way of communicating these, either supporting the participation of the central person at the Conference or representing their views to the network and practitioners if the person would prefer not to be present. Central to managing networks and situations was the confidence and skills of coordinators, particularly in enabling the central person to have their voice within a wider collaborative process.

Overall, a balanced and pragmatic approach prevailed in which it was recognised that situational and cultural factors might affect people’s willingness to engage with the approach, but that a willingness by coordinators to offer time and flexibility to take account of such factors could often (but not always) lead to agreement to participate in something that may initially seem somewhat strange or counter cultural. Similarly, issues of shame in discussing family matters with professionals could potentially be overcome by coordinators offering sensitivity and acceptance, and demonstrating cultural competence could be important. While it could be helpful to offer people the choice of working with a ‘matched’ coordinator from the same ethnic or cultural background, it was recognised that this may not always feel comfortable or appropriate. It was noted that, for some traditional families, there may be particular concerns about shame if the coordinator was to be seen as having connections with other individuals within their community, and hence a fear that other members of their community might become aware of the family situation.

**Characterising the model for FGC practice with adults: Summary of findings**

Based on the foregoing synthesis of findings from the research and practice literature, a national survey of practice, and targeted interviews with a range of stakeholders, we present, in summary form, our preliminary understanding of how the practice model for adult FGCs may best be characterised.

**Values**

Our conclusion is that FGC is a strongly values-driven process, but its underpinning values and principles are often implicit. These values demarcate a practice space with its own culture that may be significantly different from wider service cultures in social care and health, and practitioners appear committed to defending this ethos against any encroachment by hierarchical or professionally driven ways of working. Based on the evidence we obtained or reviewed, we present a summary of the key values and principles in Box 1.

**Box 1. Key Values and Principles for Family and Group Conferencing with adults.**

•   Independence of Coordinator

            ○   responsibility for process, not for what should go into the Plan

            ○   separate from support or social work roles

•   Respect for people’s strengths, abilities and preferences – and a presumption that everyone will have (some) capacity to be involved in decision making

•   Central person and their network seen as experts on their own situation

•   Central person and their network should have the right to make their own plans and decisions

•   All participants should have a voice and have the support they need so as to be heard

•   FGC processes should be tailored so as to mesh with social or cultural expectations.

**Who for, when and in what circumstances?**

From our inquiry, we conclude that FGC can be a universal offer that should not be limited to specific service user groups or offered only in response to particular sets of circumstances. More specific considerations are outlined in Box 2.

**Box 2. Family and Group Conferencing with adults: who for, when, and in what circumstances?**

FGC can be appropriate for all adult service user groups for:

- planning for support and care
- recovery and enablement
- safeguarding

It can be more effective if offered early on (such as at the point of initial request for support, or prior to hospital discharge), but it may also be useful at points of transition due to changes in circumstances or abilities.

Although the model can be applied flexibly so as to mesh better with different social and cultural norms and expectations, it cannot be assumed that it will necessarily be seen by all groups as having sufficient ‘fit’ with their preferred ways of reaching decisions.

More specific considerations:

FGC can be offered:

■ where central person lacks capacity – enlist an advocate and/or convene a Conference just for the carer network
■ where there are concerns around abuse or exploitation (as long as specific criteria around safety and responsibility are met)
■ where there are relationship issues within the network (but mediation may need to be offered first)
■ as a way of bringing together a network where existing connections may be weak or estranged
■ as part of a crisis plan – once immediate safety is assured.

Not a good option if:

■ central person feels that process would threaten their safety, or open them up to abuse or control by network member(s)
■ central person does not feel comfortable involving others in what they see as personal matters
  ■ Shame may be a potential barrier, but coordinator may be able to support them to overcome this
■ central person does not feel motivated to take part in the process – perhaps not wanting to, or feeling able to, take charge of planning their support
■ no viable basis for a network exists.

**The practice model**

Although there can be flexibility in how each stage of the process is delivered, the practice model may nevertheless be characterized as a sequence of three stages – preparation, conference, and follow-up/review–each comprising specific activities and interactions. These are summarized in Box 3.

**Table:** Box 3: Characterising the Practice Model for Family and Group Conferencing with adults

PREPARATION
• Coordinator connects with central person and each network member to explore concerns, aspirations, and preferred options ○ inclusion of widest network with whom citizen feels comfortable ○ preferences around venue, food, format for Conference ○ need for advocate? • Coordinator connects with referrer and relevant practitioners to explore concerns, options, hoped-for outcomes and any ‘non-negotiables’. • Coordinator arranges for all relevant information to be shared with all participants prior to the Conference • Coordinator sets the tone for respectful communication and acknowledgement of conflict and differences ○ agreeing some (but not too many) ground rules. **Variation:** At the outset, the coordinator supports the central person in drawing up their personal recovery plan – and then deciding what of this they want to share, as their agenda, with their network and invited practitioners.
CONFERENCE
• Participants are offered the choice of a neutral venue, such as a library or community facility. However, they may elect to hold the Conference wherever feels most comfortable to them – including the place where they live. Hybrid and online options may also be considered in order to include key people. • Start with sharing of food (or other informal activity) which brings people together on an informal basis and breaks down power hierarchies. • ‘Shared learning platform’ - Coordinator facilitates an open conversation between central person, network members, and invited practitioners. • Private Time – central person and network members devise a Plan on their own (with advocate if appropriate). Coordinator remains on hand to offer support if needed. • Feeding back and finalising the plan - central person and network members share their Plan and (either directly or via coordinator) check out with relevant practitioners and agencies (a) whether desired service inputs can be provided, and/or (b) whether any professional concerns have been satisfactorily addressed (e.g. around safeguarding). **Variations:** • In some more complex situations, the process may be split into 2 (or more) mini-Conferences held in succession. • Central person and network may invite the coordinator to sit in on Private Time and be on hand to assist at particular points in the decision-making process – particularly where there may be concerns around abuse or intimidation. • Central person and network may invite practitioner(s) to join for relevant parts of the planning process to better tailor, or coordinate, the service input
FOLLOW-UP AND REVIEW
Coordinator will follow through to support the implementation of the Plan. A minimum of one review is always offered to check how things are going, and, if appropriate, revise the Plan. However, there can be variation in the number, format and purpose of the review(s). A review can be used to: • Monitor whether plan is working, and hoped-for outcomes are being achieved • Change the Plan as needed • Celebrate successes and how people’s lives may have changed more generally Additional reviews and/or a further FGC can be offered as appropriate, particularly if circumstances change.

## Conclusion

In the forgoing summaries, we present a comprehensive understanding of the practice model for FGC in adult social care and mental health contexts in the UK, together with its underpinning values and principles and for whom it may be seen as suitable. While this characterization of the model is specific to the UK, it has potential significance internationally, where FGC is utilised in such contexts.

It is an approach that holds promise as an inclusive and network-led practice that aligns with wider policy and practice developments such as strengths-based practice, restorative practice, and local area coordination (and similar approaches to the development and mobilisation of community resources). While it is unique in privileging Private Time as the space in which plans for care and support should be made, FGC may be seen as sharing important features with other democratic and inclusive approaches such as Open Dialogue and Shared Decision Making.

Although a relatively new approach, our study suggests a wide applicability for FGC across a broad range of service contexts, and one that should be made more widely available, particularly at the point where people may be initially experiencing difficulties or when they are seeking to reestablish life in the community, for example, following a hospital stay. The approach promotes a collaborative and inclusive approach to mobilizing support around a central person, one that respects people’s autonomy and social and cultural diversity. However, crucial to the process remains the willingness and motivation of the central person to become involved, and the confidence and skills of FGC coordinators to bring network members and professionals together to devise and implement what may work best for the person at the centre.

This Paper is followed by a companion paper Part 2 ( Tew *et al.*, 2025b) which draws upon the same range of sources and uses a realist synthesis approach to elucidate how FGC may be seen to work in terms of contexts, mechanisms, and outcomes – the basis for an initial program theory for FGC in adults and mental health contexts.

## Ethics and consent

Using the HRA screening tool ( https://www.hra-decisiontools.org.uk/research/), it was determined that this study did not constitute research for which HRA approval was necessary. Ethical approval was provided by the University of Birmingham’s ethics committee (ERN-22-0818) on 09.12.2022. All participants have provided informed verbal and written consent to take part in the study.

## Data Availability

Given the small number of services that offer FGCs to adults, our survey and interview data contain information that cannot be sufficiently redacted to acceptable standards at the individual level. To request access to restricted data, please contact Sharanya Mahesh- s.n.mahesh@bham.ac.uk. Access will be provided on discretionary basis, mainly taking into account the purpose for which datasets will be utilised. Zenodo: Family and Group Conferencing (FGC) in adult social care and mental health, DOI: https://doi.org/10.5281/zenodo.14676833, ( Mahesh, 2025) This project contains the following extended data Online survey topic guide Stakeholder interviews topic guide Deliberative forum questions Data are available under the terms of the Creative Commons Attribution 4.0 International license (CC-BY 4.0).
