# Supplementary material for: What is Family and Group Conferencing for adults? Part 1: Characterising the model and methods of enquiry
Source: NIHR Open Res. 2025 Jun 6;5:20. Originally published 2025 Mar 7. [Version 2] doi: 10.3310/nihropenres.13811.2 (PMC12198747; doi:10.3310/nihropenres.13811.2)
Supplement: Peer review history

# Reviewer report: Reviewer response for version 2

**DOI:** 10.3310/nihropenres.15219.r35997

**Published:** 2025-08-28

**Authors:** Jess Harris, Joan Rapaport

**Affiliations:** 1 Health and Social Care Workforce Research Unit (HSCWRU), King's College London, London, England, UK; 2 Retired. Previously researcher at the Health and Social Care Workforce Research Unit (HSCWRU), King's College London, London, England, UK

This is an impressive document about an important topic, offering an understanding of the practice model for FGC in adult social care and mental health contexts, including the underpinning values, principles and for whom it may be suitable.

 The literature review is focused and comprehensive. The multi-mixed method approach and versatile range of research tools to capture the sensitive nature of the data is commendable. The continuous evaluation of/contribution to the study by people with lived experiences of the FGC is noteworthy.

 The source data underlying the results is not publicly available, but this is in keeping with the difficulty of retaining anonymity and ​confidentiality with particular datasets.

 Professionals will find the summary boxes containing FGC values drawn from the study, including advice regarding when and when not to use the FGC and practice model very helpful for planning and reviewing their practice.

 The theoretical frameworks are comprehensive and appropriate. The authors may wish to reflect that the potential for empowerment between the respective participants can also be reciprocal.

 The section on coercive situations would benefit from some clarification of meaning. In respect of adult services, the Mental Health Act has various means of coercion, albeit the principle of least restriction is deeply embedded in decision-making. It may be worth reflecting that there can be degrees of 'voluntariness', and people may be drawn to a 'least worse' option. 

 Regarding potential cost effectiveness, Parker et al 2018 speculate that local councils may not be very motivated to apply the adult FGC as adult services tend to cost less than those for the care of children. Savings in children's services are therefore seen to be greater and more worthwhile.

**A few specific comments on the text:**

 " *... interview topic guides... were then refined with input from our Lived Experience Advisory Panel (LEAP)... the language used was **simple **and appropriate.*" - Here the term 'simple' could be interpreted as condescending, and the term ' **clear**' might be better. 

*"It was noted that, for some **traditional **families, there may be particular concerns about shame if the coordinator was to be seen as having connections with other individuals within their community" - here the term 'traditional' is probably referring to particular religious or cultural norms that offer a poorer fit with FGC ways of thinking and working. The term 'traditional' may not be the best description of these.*

 "... *crucial to the process remains the willingness and motivation of the central person to become involved, and the confidence and skills of FGC coordinators to bring network members and professionals together to devise and implement what may work best for the person at the centre.*" - the article, rightly, offers considerable reflection on the 'central person', however it would be interesting to see findings and reflection on the extent that network members need to be prepared to contribute to the FGC plan, and whether this is a barrier to FGC success. This may be something addressed in the companion paper Part 2. 

 There are a few small errors in the text that could benefit from proofreading.

Is the work clearly and accurately presented and does it cite the current literature?

Yes

If applicable, is the statistical analysis and its interpretation appropriate?

Not applicable

Are all the source data underlying the results available to ensure full reproducibility?

No

Is the study design appropriate and is the work technically sound?

Yes

Are the conclusions drawn adequately supported by the results?

Yes

Are sufficient details of methods and analysis provided to allow replication by others?

Yes

Reviewer Expertise:

Joan Rapaport: Experience of researching FGCs and of practice as a Mental Health Social Worker.  Jess Harris: Experience of qualitative research in health and social care, including working with peer researchers.

We confirm that we have read this submission and believe that we have an appropriate level of expertise to confirm that it is of an acceptable scientific standard.

# Reviewer report: Reviewer response for version 2

**DOI:** 10.3310/nihropenres.15219.r36095

**Published:** 2025-06-25

**Authors:** Gideon de Jong

**Affiliations:** 1 Southern Cross University Faculty of Health (Ringgold ID: 110939), Lismore, New South Wales, Australia

Thank you for inviting me to advise on this research project. It has been more than ten years since I was the first (at least in the Netherlands, but to the best of my knowledge also worldwide) to receive a PhD degree on research into the process and outcomes of FGC in mental health contexts for adults. However, I have not been involved in research into FGC in recent years. Reading your article was therefore a reminder of how this process proceeds. It actually corresponds entirely with how FGCs were implemented in the Netherlands in the years that I did research on it (2008-2015). I therefore endorse the importance of: 1) an independent coordinator; 2) that FGCs can be used in any situation as long as the central person supports this decision; 3) private time; 4) and a more explicit role for coordinators to support the family group in the process of implementing and evaluating the plan (as long as resources are available for this of course).

 A number of points of attention:

- It is not customary to speak of “Holland”, but of “the Netherlands”. It would go too far to go into this in more detail, but I can tell you that about 75% of the Dutch population is not happy when their country is referred to as “Holland”.
- The article by Bredewold and Tonkens on failed conferences has a number of caveats. Firstly, it is based on a very limited number of analysed conferences, namely four. Secondly, I cannot agree with their claim that FGC would be “neoliberal”. This was a political discussion in the Netherlands that arose around the year 2008, where my colleagues and I took the position that FGC is only neoliberal if it is used as such by politicians: the model itself as it was developed (back in the 1980s in New Zealand) and the organisation in the Netherlands (“Eigen Kracht Centrale”) that introduced this model in the early 2000s do not have a neoliberal tendency at all. I myself have written an article based on over 40 case studies of an equally large number of conferences (including over 300 semi-structured interviews) that in my opinion gives a much more nuanced picture (and is based on a much richer database than Bredewold and Tonkens’s study) that addresses why conferences can apparently(!, because even if it is characterised as a failure by professionals, it can still have initiated meaningful processes) be considered a failure. De Jong G et al. 2015 (Ref 1)
- I completely agree that shame plays a central role in FGC for adults. However, I was surprised that you did not refer to the article in which I explicitly paid attention to this. The word “shame” is even included in its title: De Jong G et al. 2013 (Ref 2)
- Together with my colleagues I have written an article on how FGC compares to other models of shared decision-making (shared decision-making is quite a stretch away from FGC, much more aligned is the concept of supported decision-making, see Simmons, M. B. et al. (2017) (Ref 3) and Open Dialogue (and other models such as Flexible Assertive Outreach). Perhaps that also offers some starting points: Schout G et al. 2017 (Ref 4)

I can only support your project and hopefully it will provide an opening for a wider application of FGC in the UK.

 Best wishes,

 Gideon de Jong

Is the work clearly and accurately presented and does it cite the current literature?

Yes

If applicable, is the statistical analysis and its interpretation appropriate?

Not applicable

Are all the source data underlying the results available to ensure full reproducibility?

Yes

Is the study design appropriate and is the work technically sound?

Yes

Are the conclusions drawn adequately supported by the results?

Yes

Are sufficient details of methods and analysis provided to allow replication by others?

Yes

Reviewer Expertise:

Family Group Conferencing, Family Involvement, Community Mental Health, Assertive Outreach

I confirm that I have read this submission and believe that I have an appropriate level of expertise to confirm that it is of an acceptable scientific standard, however I have significant reservations, as outlined above.

## References

[1] : Family Group Conferencing in public mental health and social capital theory. *Journal of Social Work* .2015;15(3) : 10.1177/1468017314547675 277-296 10.1177/1468017314547675

[2] : Breaking through Marginalisation in Public Mental Health Care with Family Group Conferencing: Shame as Risk and Protective Factor. *British Journal of Social Work* .2013;43(7) : 10.1093/bjsw/bcs050 1439-1454 10.1093/bjsw/bcs050

[3] : Spot the difference: shared decision-making and supported decision-making in mental health. *Irish Journal of Psychological Medicine* .2017;34(4) : 10.1017/ipm.2017.59 275-286 10.1017/ipm.2017.59

[4] : Family Group Conferencing—Its Added Value in Mental Health Care. *Issues in Mental Health Nursing* .2017;38(6) : 10.1080/01612840.2017.1282996 480-485 10.1080/01612840.2017.1282996 28510481

# Reviewer report: Reviewer response for version 1

**DOI:** 10.3310/nihropenres.15005.r34957

**Published:** 2025-04-02

**Authors:** Kate Parkinson

**Affiliations:** 1 University of Salford, Salford, England, UK

I think this is a great article, which is well researched and referenced. 

 I have the following comments:

 Under the heading of methods, I don't think the word patient is the appropriate. All through the article, it refers to people with lived experience, which is wholly appropriate. Most people using FGCs will not be using the service because they are patients of the NHS. 

 I understand that the article was looking at the broad scope of adult FGCs and how they are used but I think some of the areas discussed lack a bit of depth as a result. For example, the use of FGCs for DVA is much more nuanced and complex than the article suggests. I think some reference to this would be helpful.

 In the paragraph beginning 'There could also be issues where members of the professional system were uncomfortable....' these are similar concerns to those that are raised in the use of FGCs in children's services and there is literature focusing on FGCs in children's service that discusses how some of these tensions can be mitigated, which could be drawn upon as evidence of what support needs to be put in place e.g the clarity of the report for the FGC, which should give families a very clear framework in which to develop their plan, the role of the independent co-ordinator in supporting families, the role of advocacy for potentially vulnerable family members and the exemption of abusive family members from the meeting. 

 I am also wondering why the following article has not been referred to and referenced- Ref: [1]

Is the work clearly and accurately presented and does it cite the current literature?

Yes

If applicable, is the statistical analysis and its interpretation appropriate?

Yes

Are all the source data underlying the results available to ensure full reproducibility?

Yes

Is the study design appropriate and is the work technically sound?

Yes

Are the conclusions drawn adequately supported by the results?

No

Are sufficient details of methods and analysis provided to allow replication by others?

Yes

Reviewer Expertise:

Family Group Conferences

I confirm that I have read this submission and believe that I have an appropriate level of expertise to confirm that it is of an acceptable scientific standard.

## References

[1] : Using family group conferences in safeguarding adults. *The Journal of Adult Protection* .2010;12(1) : 10.5042/jap.2010.0091 27-31 10.5042/jap.2010.0091

# Response

**Published:** 2025-05-20

**Authors:** Sharanya Mahesh

**Affiliations:** Social Work and Social Care, University of Birmingham, Birmingham, England, UK

Many thanks for your constructive reviews on our article. We have taken your feedback into consideration and addressed the following –

- We have taken a balanced approach and added further detail regarding FGC outcomes for children in the introduction.
- We have drawn parallels to other approaches such as Shared Decision Making and Open Dialogue in the introduction.
- More detail is provided regarding the use of FGC with individuals experiencing domestic violence and abuse. We have also included the suggested article (Tapper L: Using family group conferences in safeguarding adults) to strengthen our understanding about FGC and DVA.

Other feedback addressed

**Independent coordinator’s involvement post FGC conference**: In our study, it was consistently evidenced that coordinators continue to be a part of the process after the conference. It is the coordinator who liaises with other professionals (including the referrer) to confirm the ‘plan’ and check the feasibility of implementing the ‘plan’. The coordinator continues to be involved until after the review (s) have been undertaken and typically withdraws involvement at this stage to reduce dependencies.

**Professional systems being uncomfortable with handing over power to the central person and their network:** While there may be lessons we can learn from children’s FGC, we deliberately chose not to include children’s literature in our research sample on the basis that the context and positioning of professionals are significantly different. For example, in children’s FGC, it can be the professionals’ report for the FGC that gives families a clear framework to help develop their plan but this is not the case in adults unless there are safeguarding concerns.

# Reviewer report: Reviewer response for version 1

**DOI:** 10.3310/nihropenres.15005.r34960

**Published:** 2025-03-26

**Authors:** Shulamit Ramon

**Affiliations:** 1 University of Hertfordshire, Hatfield, England, UK

The authors assume that the use of FGC is unproblematic in the context of child care. This is not born out by research on this issue. See research published by Nuromatov et al 2020 (Cardiff University) which states that outcomes of the application of FGC in child care are inconclusive.

 FGC is a form of shared decision making, by the participants in the FGC meetings, aimed for the decisions made by the FGC to be followed up by the Key Social Worker who initiated the request for the process to take place. Yet SDM is not mentioned in this otherwise useful and good quality research. 

 Although the Family Open Dialogue is a form of group therapy, it is also one in which family members are actively involved alongside therapists from the outset. This approach has been well researched internationally, and deserves to be looked at in the context of FGC. 

 The text seems to suggest that the independent co-ordinator continues to be involved also at the phase of post the initial FGC meeting. To the best of my knowledge this is not the case, as following the FGC decision making meeting it is the key social worker who initiated the referral continues to work with the family and the index client, and not the co-ordinator. 

Is the work clearly and accurately presented and does it cite the current literature?

Yes

If applicable, is the statistical analysis and its interpretation appropriate?

Not applicable

Are all the source data underlying the results available to ensure full reproducibility?

Yes

Is the study design appropriate and is the work technically sound?

Partly

Are the conclusions drawn adequately supported by the results?

Partly

Are sufficient details of methods and analysis provided to allow replication by others?

Yes

Reviewer Expertise:

Different forms of shared decision making in mental health, including FGC Research on FGC and on involving people with lived experience of mental ill health in interventions and in research, as well as actively involving EbEs (experts by experience) in education and research.

I confirm that I have read this submission and believe that I have an appropriate level of expertise to confirm that it is of an acceptable scientific standard, however I have significant reservations, as outlined above.
